# Transdisciplinary care in practice: reflections from the Council of Care

**DOI:** 10.1007/s42532-025-00229-w

**Published:** 2025-10-27

**Authors:** Adriana Ressiore C., Gabriela De La Rosa

**Affiliations:** 1 Wageningen University and Research, De Leeuwenborch, Hollandseweg 1, 6706 KN Wageningen, The Netherlands; 2 National Institute of Science and Technology in Interdisciplinary and Transdisciplinary Studies in Ecology and Evolution (INCT IN-TREE), Bahia, Brazil

**Keywords:** Art-based methods, Care, Collective, Empathy, Participatory action research, Transdisciplinarity, Trust, More-than-human

## Abstract

Environmental decision-making is often still limited in its capacity to fully engage with social sciences and local communities. This results in the reinforcement of top-down approaches that exclude diverse perspectives. This paper proposes an arts-based method rooted in transdisciplinarity and participatory action research: the Council of Care, developed and implemented in Brazil and the Netherlands to foster inclusive dialogue and collective engagement. Drawing from the Council of All Beings and the Theater of the Oppressed, it uses role-playing and empathetic engagement to amplify marginalized voices and shift focus beyond human-centric narratives. The method has been applied across educational, community, and policy settings, promoting deeper awareness of the interconnectedness and interdependence among all beings. Our reflections highlight both the challenges and opportunities in using the Council of Care to support more caring and collaborative decision-making and socio-ecological research practices. By highlighting diverse forms of human and nonhuman agency, this approach encourages a more participatory future, with creative practices that aim to include human and nonhuman actors in decision-making processes.

**Supplementary Information:**

The online version contains supplementary material available at 10.1007/s42532-025-00229-w.

## Questions and methods for caring and transdisciplinary collaboration

There is growing attention to engaging Indigenous and local knowledge in biodiversity and environmental international politics (Pascual et al. 2021, p. 569; Díaz-Reviriego et al. 2019, p. 457; IPBES 2019, pp. XX-XXI; CBD 1992, p. 3). However, decision-making processes that aim to address socio-ecological challenges still often exclude insights and knowledge(s) from local communities and other marginalized actors (Albuquerque et al. 2021, p. 2; Liu et al. 2018, p.​​469). Transdisciplinarity has been presented as an alternative to narrow disciplinary approaches, fostering dialogue between different knowledge and sociocultural systems (Scholz and Steiner 2015, p. 522; Popa et al. 2015, p. 46; Bammer et al. 2020, p. 6; Lang et al. 2012, p. 26; Ludwig and Boogaard 2021, p. 20). In this article, transdisciplinarity and care serve as our overarching conceptual framework.

We approach transdisciplinarity as a space for learning and co-production across knowledge systems, while care informs how we attend to the relational, affective, collective, and political dimensions of collaboration (Staffa et al. 2022, p. 49; Sellberg et al. 2021, p. 301; Diver et al. 2024, pp. 1–3 of 12; Puig de la Bellacasa 2017, p. 4; Bartos 2018, p. 67). Participatory action research (PAR) and more-than-human participatory approaches support this transdisciplinary orientation, helping us understand how inclusive and situated decision-making can be fostered in practice (Ludwig and El-Hani 2020, pp. 3–4; De la Rosa et al. 2024, pp. 9–10 of 12; Bastian et al. 2017, p. 5). However, bringing different knowledge systems and perspectives together is a challenge. The difficulty lies in how the confluence of various types of knowledge can be achieved (Bispo dos Santos 2023, p. 4). Addressing this challenge requires methods that allow participants to share vulnerabilities, desires, experiences, and ideas for the future.

The research presented in this article asks: What kinds of methods can support the creation of spaces where diverse human and nonhuman participants can express vulnerabilities, desires, and ideas for the future, and participate meaningfully in decision-making? To unpack this broad inquiry, we ask: (1) What are the methodological requirements for co-creating such spaces with care and sensitivity to context? (2) What are the potentials and challenges of this method when it is implemented in diverse decision-making contexts? (3) How can arts-based and embodied methods such as the Council of Care support transdisciplinary and more-than-human engagement?

These questions emerged from collaborations and learning with human and nonhuman communities, which are often excluded from dominant decision-making processes. We also aim to reflect on the use of the Council of Care as a participatory method. In this sense, we follow Xiang’s (2023, pp. 364–365) notion of practice-relevant research as that which offers tools and insights that are directly relevant, actionable, and inspiring for real-world practitioners.

To address our research questions, we developed the Council of Care, an arts-based method influenced by multiple practical iterations and theoretical approaches, including the Council of All Beings (Seed et al. 1988), Theater of the Oppressed (Boal 2021), the work on Matters of Care (Puig de la Bellacasa 2017),^1^ and the Parliament of Things (Latour 1991, 2018). During the Council of Care, we invite the participants to put themselves in someone else's shoes, whether human or nonhuman beings. This practice challenges modern human–nature dualities and amplifies nondominant voices (Carvalho and Riquito 2022, p. 38; Descola 2013, p. 28 of 251). With this method, we aimed to create a safe space for participants to embody other beings and engage in role-play—speaking from their perspectives and considering the others’ points of view. We strived to create an appropriate setting and instigate the participants to play with their imagination through embodiment and connection with their senses (Pearson et al. 2018, pp. 3–9). This involved presenting a real-life situation to discuss and work on collectively, and facilitating dialogue in a mock decision-making process, with the hope of reaching decisions and actionable plans. Through this process, we began to understand diverse worldviews and desires, which in turn helped us draw insights into engagement, embodiment, participation, collective decision-making, and internal conflicts.

This research is intended for scholars, practitioners, and community partners engaged in participatory and transdisciplinary work, particularly in contexts where environmental and social challenges intersect. Developed through empirical work in Brazil and the Netherlands, the Council of Care is not a universal approach, but rather a flexible method that invites context-specific adaptation across diverse institutional and socio-ecological settings. In the following sections, we introduce this arts-based method, informed by participatory action research, and offer a critical and empirical reflection on its challenges and potentials. To further our investigation, we present the theoretical framework, positionality, context, and methodological foundations that supported the development of the Council of Care. We then describe the structure of the method and its implementation across various settings. Our analysis, grounded in empirical findings, highlights the method’s transdisciplinary and caring insights as evidenced in its application within socio-ecological contexts. In the discussion, we offer a critical reflection on the method’s challenges and potentials for participatory decision-making in such contexts. We conclude with reflections on the transformative potentials of this method and its implications for building participatory and caring futures.

## Literature and methodological foundations

### Literature foundations

In this section, through an integrative literature review (Snyder 2019, pp. 335–336), we position the Council of Care within existing PAR, more-than-human methodologies, and Arts-based literature, highlighting its unique contributions and areas of limitation when considering existing approaches.

#### Participatory action research and other participatory planning approaches

In our transdisciplinary approach, PAR serves as a key methodological foundation. PAR is a continuous and reflexive process involving partnerships between researchers, community members, and other societal actors to collaboratively identify and address issues of local importance (Kindon et al. 2007, pp. 1–19; Smith et al. 2010, pp. 1–3; Glassman and Erdem 2014, pp. 207–208). Rather than treating communities as subjects of research, PAR emphasizes collaborative knowledge creation oriented toward social transformation.

Rooted in Latin American perspectives of the pedagogy of the oppressed (Freire 1970, 1982; Fals-Borda 1991) and feminist critiques (Smith et al. 2010; Harcourt et al. 2022), PAR focuses on fostering agency, dialogue, and the dismantling of oppressive structures. PAR has various origins (Smith et al. 2010, pp. 407–408); however, this article focuses on the agency of the often-oppressed in their struggles to free themselves from their oppressors. The struggle of the colonized to resist the narrow structures and expectations established by those who colonized them, and the struggle of those who are rendered invisible or subordinated by the more powerful (Glassman and Erdem 2014, p. 207; Brear and Tsotetsi 2022, pp. 813–830). Two aspects are particularly central to our methodological development and implementation: (1) the research process involves continuous dialogue between facilitators and community members, and (2) understanding community relations and social problems requires a rigorous, interactive, and cyclical process of discovery, reflection, and realization (Glassman and Erdem 2014, p. 209).

Political decisions are commonly taken with a top-down approach, failing to consider the agency of multiple beings (Lal et al. 2002, pp. 3–4). Through a transdisciplinary and PAR lens, it is possible to rethink and transform plural decision-making processes into something genuinely collaborative and co-developed with diverse societal actors, including those most commonly excluded from political spaces. Relating the decision-making process to PAR has been discussed in several studies (Senecah 2004, pp. 14–16; Milich et al. 2021, pp. 1–15; Perz et al. 2022, pp. 5–7; Khanyari et al. 2023, pp. 1–14), such as that developed by Lal et al. (2002), who highlight what they call an adaptive decision-making process (ADP). Their approach to ADP stresses the importance of a reflexive process, which aims to co-produce management strategies and foster a sense of belonging among participants involved. This article follows these authors in aiming for a process that recognizes multiple actors “who have different values and knowledge systems and use multiple paradigms. It acknowledges the need for a dialectic decision-making process supported by rigorous single- and multidisciplinary research" (Lal et al. 2002, p. 4).

Participatory planning more broadly encompasses a rich body of theory and practice focused on collaboration, trust, collective action, and democratic deliberation. Influential contributions from collaborative and deliberative planning theorists—such as Healey (1997, pp. 5–10), Innes and Booher (2004, pp. 419–423), and Reed (2008, pp. 2419–2421)—have investigated how inclusive planning can address power dynamics and generate more just outcomes. We also learn from the multidisciplinarity of participatory methods (Bacon et al. 2005; Chevalier and Buckles 2013, pp. 15–18; Muhammad et al. 2015, pp. 1045–1063; Schinke and Blodgett 2016). Deliberative tools such as citizens' assemblies and consensus conferences emphasize structured dialogue for decision-making (Fung 2006, pp. 20–21), while participatory budgeting foregrounds democratic control over resource allocation (Wampler 2012, pp. 1–2). Recent work has also experimented with democracy labs, which foster citizen-centered, co-created democratic innovations in sustainability transitions (Campos et al. 2024, pp. 367–380).

While these approaches have helped illuminate the limitations and ambitions of participatory practice, this article focuses on a different set of methodological concerns (see 2.1.2). In particular, we draw from more-than-human, PAR, and arts-based methods that question anthropocentric assumptions and expand the notion of who—or what—participates in collective processes (Seed et al. 1988; Boal 2005; Puig de la Bellacasa 2017). The following section discusses how these influences have informed the development of the Council of Care as a situated method shaped through practice, arts, reflection, and care.

#### More-than-human and arts-based approaches and inspirations

The Council of Care was inspired by particularly arts-based traditions and more-than-human participatory experiments. It draws inspiration from the Council of All Beings (Seed et al. 1988; Kozak 1995), the Parliament of Things (Latour 2018, pp. 47–64), Matters of Care (Puig de la Bellacasa 2017), and arts-based engagement practices (Pearson et al. 2018, pp. 3–14) such as Theater of the Oppressed (Boal 2005, 2021). Unlike deliberative methods focused on consensus, our Council of Care aims to foster relational engagement, empathy, and a sense of care that tries to include both human and nonhuman actors.

The Council of All Beings is based on Joanna Macy’s work and Seed et al.'s (1988) invites participants into ritual stages of Mourning,^2^ Remembering,^3^ Council Gathering,^4^ and Work for The Planet.^5^ The first three stages investigate participants' experiences, usually about environmental destruction. The final stage aims to foster future imagination and adaptability on this planet. This practice is inspired by philosophies of deep ecology (Kozak 1995; Naess 2005). The gathering “aims to enhance the commitment and resources of people to care for and protect the planet Earth” (Kozak 1995, p. 42).

While our method is inspired by efforts to include nonhumans in environmental discussions, it follows a distinct structure. Rather than adopting the traditional Council of All Beings format, our approach is designed to simulate a more formal policy decision-making process. Unlike the Council of All Beings, which includes stages of mourning and remembering, our method does not go through these stages. Unlike some versions of the Council of All Beings, which involve participants making masks, in the Council of Care, organizers provide badges. See the complete structure of our method in Annex 2. These changes were not only practical adaptations but also reflected a deliberate attempt to move from symbolic ritual to a decision-making simulation, aligned with transdisciplinary processes in socio-ecological practices.

Our adaptations are mindful of critiques of deep ecology’s universalist ethic, recognizing that it may not account for the diverse ways different cultures relate to nature (Plumwood 2002, pp. 62–65; Descola 2013, p. 10). Our positionalities and academic backgrounds (see 2.2) enabled us to ground the Council of Care in a Brazilian context as well as local and Indigenous perspectives (Bispo dos Santos 2023; Krenak 2020). Through its development and implementation,^6^ we aimed to surface the notion that humans are merely one more being in the world. We endeavored to create a space where, through imagination and practice, nonhumans could advocate for themselves. In that sense, Indigenous leader Ailton Krenak argues, "I do not perceive that there's anything that is not nature. The cosmos is nature. Everything I can think of is nature" (2020, p. 5). Thus, the Council of Care emphasizes situated relationalities and nondualistic understandings of the world (Ressiore et al. 2024b, p. 16).

The performative and participatory dimensions of the Council of Care are strongly influenced by the Theater of the Oppressed (Boal 2005, 2021) which is a “set of dramatic techniques whose purpose is to bring to light systemic exploitation and oppression within common situations and to allow spectators to become actors” (Coudray 2017). By transforming the spectator into a spect-actor, Boal defended that when becoming actors, the oppressed (spectators) could also become political actors in everyday life (Coudray 2017; Boal 2005). In other words, this practice aims to amplify the voices of those who have less power and are marginalized (Boal 2005; Coudray 2017).

Another methodological and theoretical influence was the Parliament of Things, which is broadly understood as “an alternative to the Modern Constitution, bringing nonhumans into the sphere of political deliberation by resorting to human representatives/mediators” (Carvalho and Riquito 2022, p. 42; Latour 1991, p. 144). Similarities and divergences between our methodology and this specific one were also identified. In the conventional structures of political deliberation, the Parliament of Things also aims to incorporate diverse voices into decision-making processes, a process analogous to the Council of Care. However, as indicated by its name, the Parliament of Things includes “things”, which is also where our methods diverge. As Latour proposed and others have implemented, there can also be inanimate “quasi-objects”, while the Council of Care aimed at only living beings to participate.

Moreover, Latour’s work also influenced Puig de la Bellacasa’s book, *Matters of Care* (2017), which has informed our understanding of more-than-human care (Ressiore et al. 2024b, pp. 3–4) and inspired our imagination and practices regarding the possibility of bringing oppressed and nonhuman entities into decision-making tables. Care is often understood as “everything that we do to maintain, continue, and repair our “world” so that we can live in it as well as possible. That world includes our bodies, ourselves, and our environment, all of which we seek to interweave in a complex, life-sustaining web” (see Tronto 2013, p. 19; Fisher and Tronto 1990, p. 40). With such definition in mind, Puig de la Bellacasa challenges anthropocentric notions of responsibility by emphasizing that "care is a human trouble, but this does not make of care a human-only matter" (2017, p. 2).

Centering the Council^7^ around care has two facets: practice and theory which relates to the Council’s steps (Annex 2) and considers an extensive literature of feminist thinkers who extensively debated care as a transformative lens and practice (Care et al. 2021, pp. 703–704; Conradi 2015, p. 125; Moriggi et al. 2020, p. 2; Staffa et al. 2022, p. 49; Sellberg et al. 2021, p. 293; Ressiore et al 2024b, p. 18) while also engaging with Brazilian thinkers (Krenak 2020; Bispo dos Santos 2023; Freire 1970).

The structure of the Council of Care was further shaped by practical resources, such as the toolkit "Arts-Based Methods for Transformative Engagement" (Pearson et al. 2018), which provided methodological guidance on designing participatory steps that foster creative engagement, embodiment, and dialogue.

Our aim for situated implementations (Haraway 1988, p. 581) is influenced by our Brazilian roots and the context of our research; however, we also believe that with appropriate adaptations (see 2.4) and critical reflections, the relevance and applicability of the Council of Care could extend to other international contexts. By aiming for a caring decision-making process that contributes to socio-ecological research, we consider that thinking and acting with care before, during, and after implementation allows us to be more attentive to needs. It has also highlighted aspects of collective care and prompted empathy.

### Researcher positionalities

We are Brazilian researchers who developed the Council of Care method and co-authored this article. Adriana Ressiore C., a social scientist working through transdisciplinary approaches, focuses on human–nonhuman relations and care theories for biodiversity conservation. Gabriela De La Rosa, a biologist trained in inter-transdisciplinarity, works on participatory methods for environmental conservation. Both are part of the Global Epistemologies and Ontologies (GEOS) research project, which supports inter-transdisciplinary research in Latin America, Europe, and Africa. The collaboration between the two authors began in the Netherlands at the start of their PhD studies, and it strengthened during fieldwork in a Brazilian fishing village (see below for more details). The Council’s pilot was developed in early 2021 during a PhD course^8^ to investigate its fieldwork applicability. Since then, we have continued its implementation (see Sect. 3.2).

Our diverse experiences shaped the Council’s creation: Adriana drew inspiration from Model United Nations^9^ and her interest in nonhuman decision-making, whereas Gabriela brought expertise in community-based conservation. We are committed to addressing power inequalities through participatory, arts-based, and reflexive research practices.

### Study contexts

**Fig. 1:**
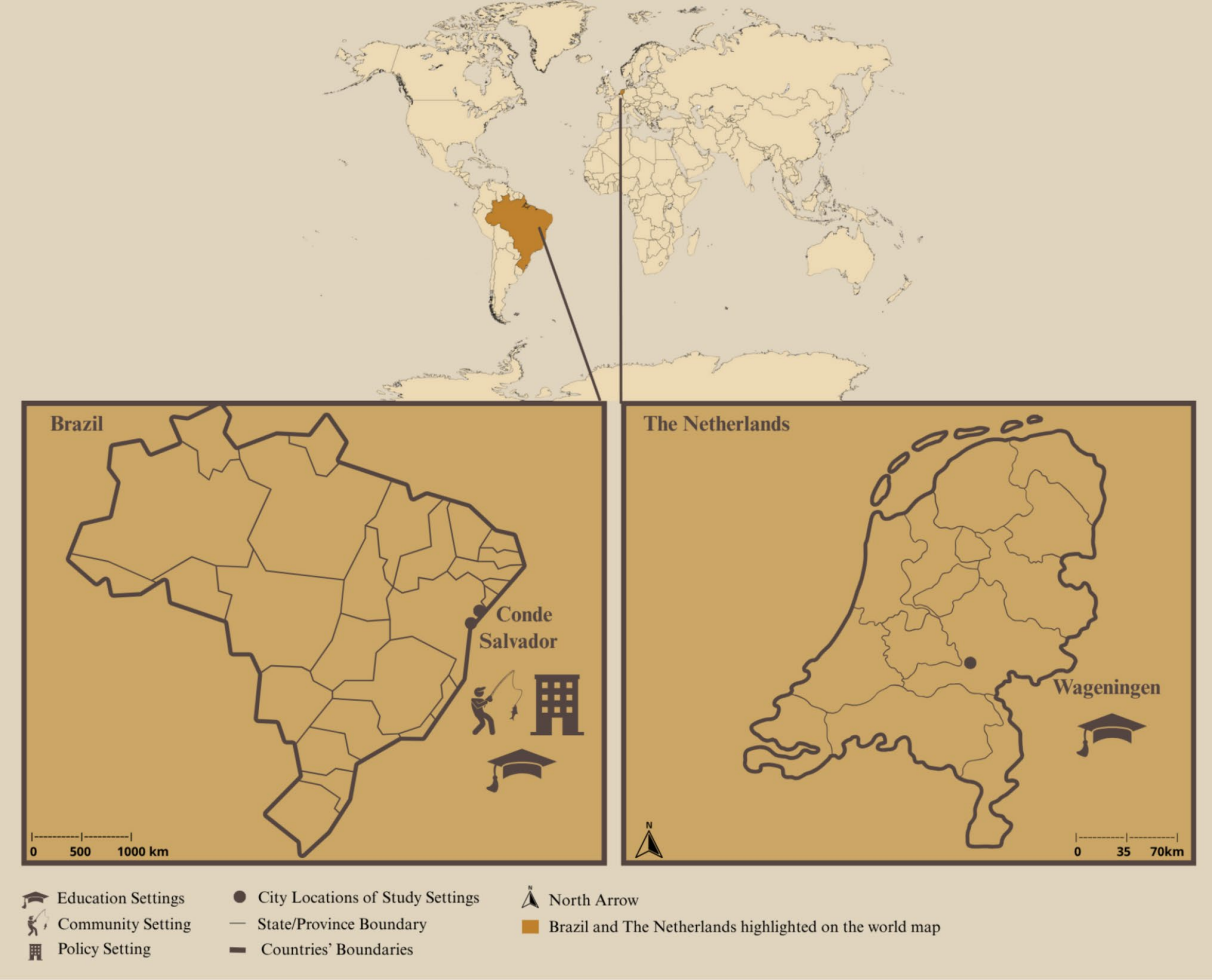
Maps showing the locations and key contexts involved in the research. In Brazil, the three icons represent the interconnected contexts of the fishing community of Siribinha (community setting), located within the municipality of Conde; local policy-makers in Conde (policy setting); and the Federal University of Bahia (UFBA, in Portuguese) in Salvador (Education Setting). In the Netherlands, the round icon marks the education and academic setting of Wageningen University and Research (Education Setting). Image created by the authors using Canva and MapChart

This study draws on empirical research conducted in three distinct yet interconnected contexts: Conde, Siribinha (a local fishing community within the municipality of Conde), and interdisciplinary academic environments in Brazil and the Netherlands. These settings reflect the transnational and transdisciplinary character of the research process, which involved collaboration with diverse actors across cultural, geographical, and institutional contexts. Figure 1 offers a visual representation of these locations and the different actors involved in each. Engaging with these diverse contexts allowed for the testing and adaptation of the Council of Care method in real-world environments marked by different knowledge systems, power dynamics, and socio-ecological priorities.

#### Siribinha and the Municipality of Conde, Bahia, Brazil (local community setting)

Siribinha is an artisanal fishing village with approximately 500 inhabitants situated in the municipality of Conde, Bahia, Brazil (Bahia 2003). The local economy is based on coconut plantations, cattle raising, and, particularly in coastal communities, fishing and low- to middle-income tourism (Tng et al. 2021, p. 6; Renck et al. 2022, pp. 635–636; Demasi 2023, pp. 10–11). Throughout the years, deep ties have developed between the Federal University of Bahia and the village through an educational and conservation project (Bollettin et al. 2023, p. 48). In 2016, a transdisciplinary team of researchers from diverse academic backgrounds and both the Global South and North initiated their activities in Siribinha. Initially, motivated by the village's preserved artisanal fishing culture and well-conserved environment, this collaboration gave rise to ongoing participatory research practices. Many of the insights presented in this paper are grounded in these long-term relationships and collaborations with community members.

In Siribinha, the fieldwork was grounded in a trust-based collaboration that unfolded across three interconnected spaces: the fishing community itself, where everyday life and local ecological knowledge helped shape the research questions; the Municipal Secretariat of Environment, where environmental policy-makers navigated tensions between conservation goals and local livelihoods; and academic partners based in multiple universities, who took part in exchanges and co-learning processes. These engagements extended beyond formal research activities and were embedded in shared experiences, informal conversations, and collective reflections on coastal changes and the politics of knowledge and care.

#### Conde’s municipal secretariat of environment and economic development (local policy setting)

The Conde Municipal Secretariat of Environment and Economic Development (Secretaria Municipal de Meio Ambiente e Desenvolvimento Econômico de Conde) is part of the political and organizational structure of the municipality. It functions as a supervisory, constructive, and deliberative body. Its roles include managing administrative procedures and drafting environmental legislation (Conde, n.d. 2025). Located in the municipality of Conde, in the state of Bahia, Northeast Brazil, the secretariat is responsible for implementing local policies related to environmental conservation, land use, sustainable development, and economic initiatives. It plays a crucial role in translating environmental policies into local actions, particularly in ecologically sensitive areas such as the coastal community of Siribinha.

#### Academic and educational institutions in Brazil and The Netherlands (education setting)

The Education Setting included scholars, students, and practitioners from various fields, career stages, and national backgrounds, each with differing or no connections to Siribinha. In Brazil, the Council of Care was primarily implemented at the Federal University of Bahia (UFBA), located in Salvador, particularly within the Biology and Ecology departments, with participation from students, professors, and researchers. In the Netherlands, the method was applied at Wageningen University and Research (WUR), in classroom settings and focus groups organized by the authors and collaborators. The participants in the Dutch context came from a diverse range of transdisciplinary backgrounds, including master's students, PhD candidates, postdoctoral researchers, professors, and lecturers.

These three study contexts—community, policy, and academic—formed the basis for the Council of Care’s implementation and analysis, each contributing unique insights into its potential and challenges in fostering inclusive, caring, and participatory decision-making processes. To achieve these goals, we also recognized the need to integrate participatory methods.

### Integrating participatory methods

While the Council of Care is a method in itself, we believe that PAR-informed research requires methodological diversity. To harness creativity and draw on diverse methods for understanding the limitations and potential of each context, methodological integration and adaptability became crucial for implementing the Council in specific settings and issue-specific contexts.

Methods such as participant observation (Wästerfors 2018, p. 314; Flick 2009, pp. 1–17; Jackson 1983, pp. 39–40) and semi-structured interviews (Roulston and Choi 2018, p. 233; Hesse-Biber 2013, p. 284) have been employed. Conducting participant observation was essential to grasp the contextual methodological potential, effectiveness, difficulties, and challenges. We adopt a feminist approach to participant observation in this research, emphasizing engagement with the everyday experiences, relationships, and affective dimensions of a community or group. One way to reach that is by immersing ourselves in the community and sharing our vulnerabilities, desires, and fears. This is not a linear or objective “technique”, and its subjective effectiveness relies on a combination with other methods, such as interviews, workshops, field diaries, and work groups.

Integrating methods is important for developing and adapting the methodology to the context and the needs. For example, semi-structured interviews were used within the Local Community and Policy settings to understand whether the methodology was fitting the context, necessities, and needs, as well as to confirm or contradict some of the perceptions mediators had during the Council’s implementation. These findings enabled us to gain a deeper understanding of how community members felt during the process and to determine whether our observations during the Council of Care regarding the community’s inner dynamics and perceptions aligned with their own interpretation. For this purpose, during fieldwork, we worked on improving and adapting the interview questions and our research goals, as well as reflecting upon each step of the Council of Care.

These methodological integrations shaped not only how we conducted fieldwork but also how we came to understand and develop the Council of Care itself, as a method that must remain open, flexible, and responsive to each setting in which it is implemented. The Council of Care was not initially designed with scalability or multi-context adaptability in mind (Tsing 2012, p. 507). Instead, it emerged through an experimental and relational process rooted in arts-based methods, curiosity, and field-based learning. As we implemented and reflected on the method across different settings—academic, community, and policy—we understand that its strength lies not in offering a standardized model, but in its capacity to be sensitively adapted through engagement and collaboration. We aimed to develop a method that is flexible in its relational nature, relying on trust-building, attentiveness to context, and the integration of other participatory tools. This helped mediators to understand local needs, power relations, and dynamics of care or carelessness. In this sense, we see the Council of Care not as a universally replicable method, but as a framework that aims for situated co-creation. Its cross-regional potential lies not in universality, but in its invitation to foster situated adaptations to individual decision-making settings. This does not mean that the method is inapplicable elsewhere, but rather that it must be accompanied by other PAR engagements, especially when working with local communities or in policy settings.

## The Council of Care: method development

### Structure of the council of care

**Fig. 2:**
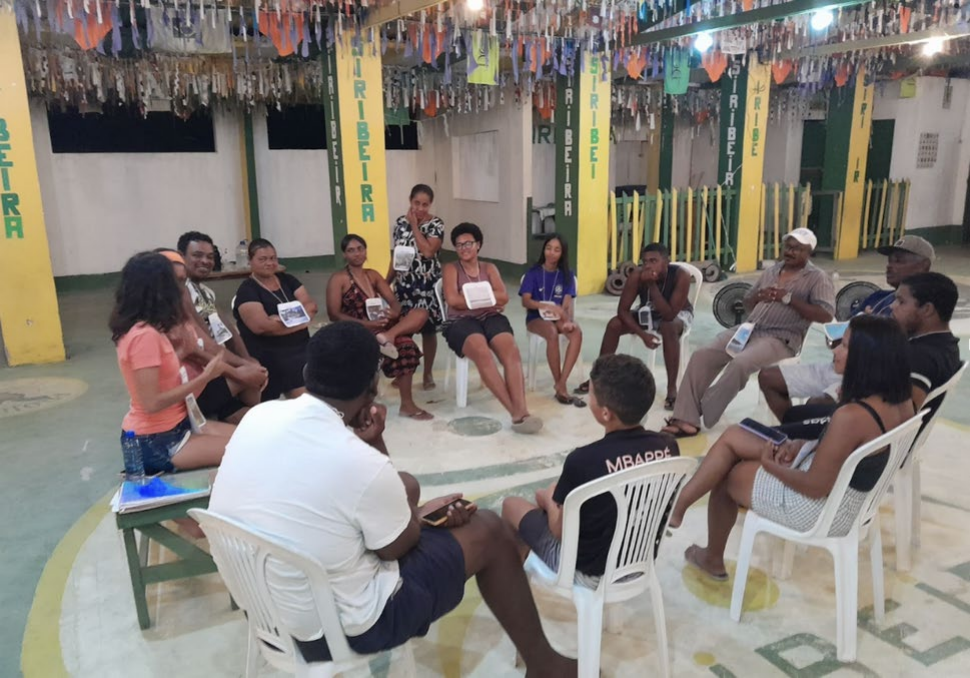
Implementation 6: Community Setting. Photograph by Gabriela De La Rosa, reproduced with permission

**Fig. 3:**
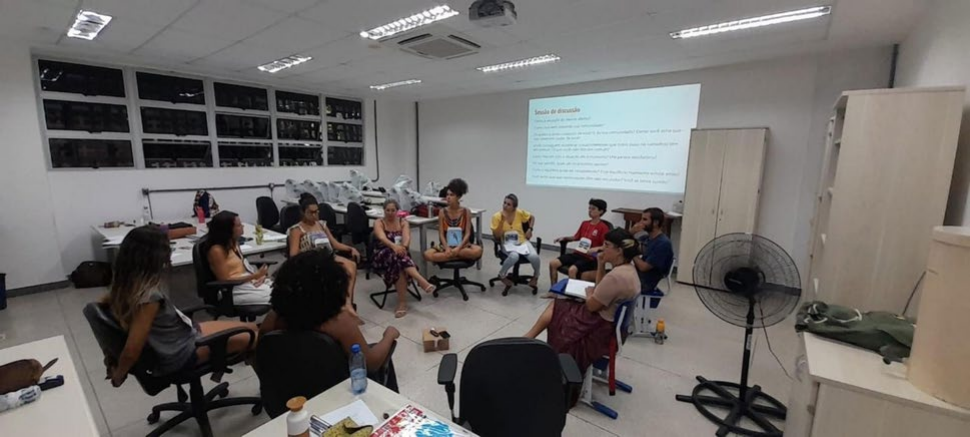
Implementation 8: Education Setting. Photograph by Gabriela De La Rosa, reproduced with permission

The several theoretical and practical examples mentioned above allowed us to create the main structure of the Council of Care. The council’s structure comprises six main stages: check-in, contextualization, embodiment, council meeting (Figs. 2 and 3), check-out, and harvest (explained in detail in Annex 2). This structure has been consistent across all implementations, with adaptations made to suit the specific setting, issues, and participants. Adapting the structure became essential for making the process more attentive and responsive to participants' needs (Care et al. 2021, p. 705) and the contexts (Sect. 2.3). These adaptations were informed through meetings between the two mediators (the authors of this article), who reflected on participants’ feedback from earlier implementations and observed emerging needs during the process.

As a methodological contribution, we aim to share insights from our experience, supporting others interested in simulating decision-making processes involving both human and nonhuman actors. In Annex 2, we suggest the time taken to implement each step; however, adjustments to the duration of each step are often necessary, depending on the setting and number of participants.

### Implementations of the council of care

**Fig. 4:**
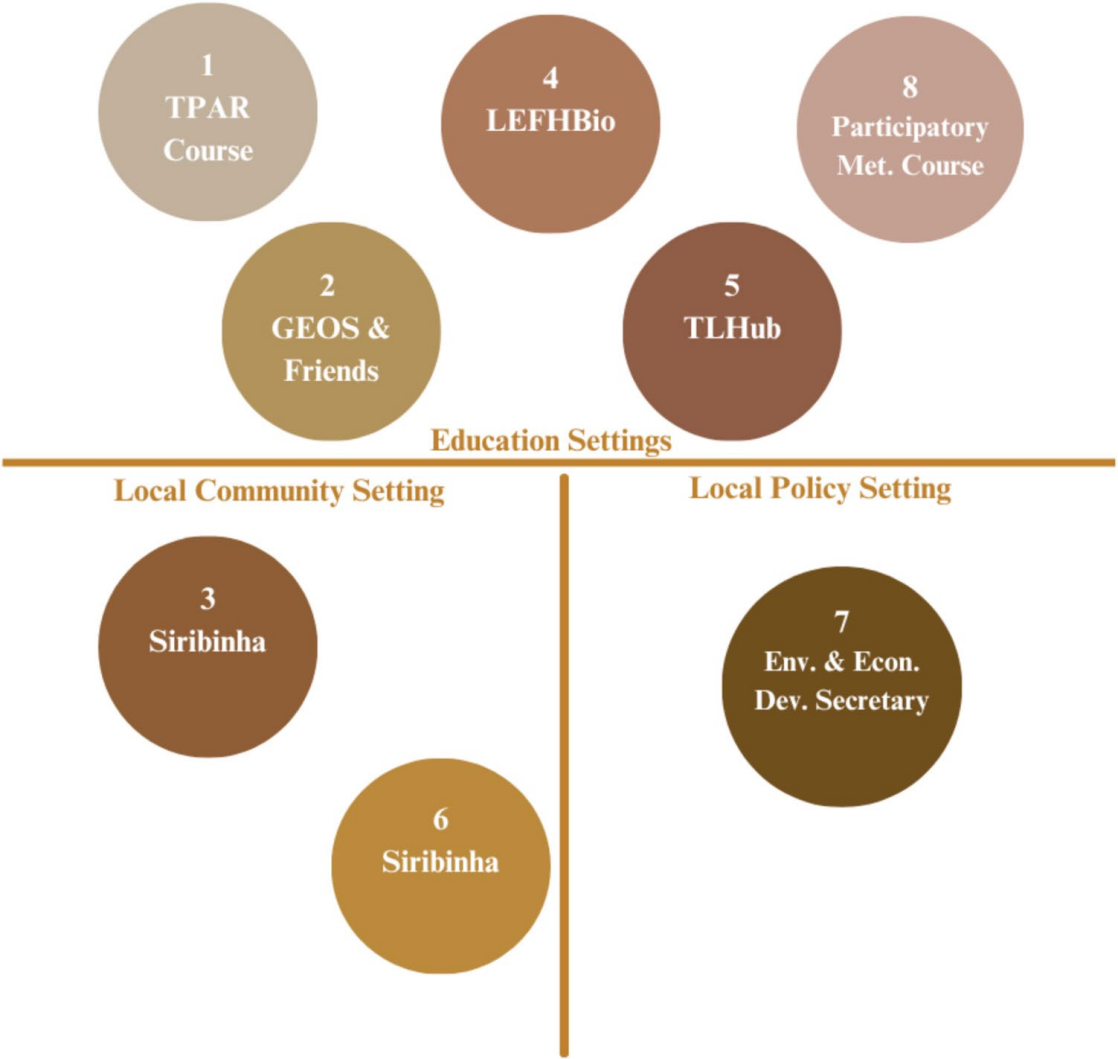
Implementation Settings. This figure summarizes the eight implementations we analyze and discuss throughout the article. We refer to them using the numbers shown in the figure, which reflect the chronological order in which they were applied. The implementations are clustered into the following groups: "Education Setting," which contains the times we implemented the Council of Care in academic, research, and classroom settings in the Netherlands and Brazil; "Local Community Setting", which refers to the occasions when the method was applied in the fishing village of Siribinha, as part of fieldwork focus group activities; and "Local Policy Setting", which includes the instance where it was applied with the employees of the Conde’s Municipal Secretariat of Environment and Economic Development, Brazil. Figure created by Adriana Ressiore using Canva

We implemented the Council of Care on more than ten occasions; however, this study focuses on the eight implementations (Fig. 4) for which we obtained formal consent to document, analyze, and publish data.

These implementations—conducted in both Brazil and the Netherlands—differed in the number of participants, physical setting, facilitation team, and context-specific adaptations. Despite this diversity, several research goals permeated all of them: (i) our individual research objectives to develop and improve the method; (ii) investigate the possibilities of a method that works to reflect on the role of the nonhuman in a decision-making process; and (iii) to share a method that can spark interest in arts-based and participatory approaches to collective and caring decision-making for complex socio-ecological contexts.

Among the different settings, Siribinha played a key role in shaping the method through multiple implementations. Many interactions with the community contributed to the adaptations (see Sect. 2.4). They played a crucial role in shaping the Council of Care as a method for collectively reflecting on some local issues (see Annex 1).

The Local Policy Setting involved a local policy-making secretariat composed of individuals from the municipality of Conde, including some from Siribinha and nearby areas. The secretariat's employees have diverse academic backgrounds and varying relationships with Siribinha, shaped by local power dynamics. Most hold higher educational degrees and, to some extent, have more economic resources, institutional power, and voice than community members do.

The Education Setting, as mentioned above, included scholars from various fields, career stages, and countries, each with a different or no connection to Siribinha. We implemented the Council of Care more frequently in this setting because of the wide range of interests and opportunities for developing and improving the method. Collaborating with colleagues and students from Brazil and the Netherlands provided diverse responses, which helped to refine the method and promote art-based approaches. These exchanges encouraged us to consider how the method could be adapted for use across various settings and participant groups.

## Data collection and analysis

Data collection and analysis took place during the creation and refinement of the Council of Care as a methodological process. Inspired by PAR cycles of action and reflection (Cahill 2007, p. 181), we integrated and combined several approaches in this circular research practice. During each implementation, the mediators took notes and held debriefing meetings to gather insights—these became the core data for analysis. These notes supported reflection, method refinement, and insight generation. While all the sessions were audio- or video-recorded, the quality of some recordings was limited due to the number of participants, distance from the microphones, and open-air environments. Education Settings were usually in a more controlled environment, allowing us to take more notes (in comparison to the other settings) and write down quotes during the sessions. The notes and recordings were revisited to gather insights, mainly from the check-in, Council of Care meeting, check-out, and feedback and harvest steps (Annex 2).

Data collection spanned approximately three years, whereas analysis took place over several months through recurring meetings and iterative revision. This process helped refine the article’s objectives. The different academic backgrounds of both authors provided a solid foundation for our critical perspectives on each other’s interpretations and discussions, which were essential for creating a cohesive storyline and data analysis. PAR also teaches that relevant data often includes dissonance and productive tensions (Cahill 2007, p. 187; Poliseli and Leite 2021, pp. 79–80). Addressing these tensions requires time and multiple methods (Pearson et al. 2018, p. 11) to facilitate joint reflection and interpretation. For this article, we focused on observations that both authors identified in two or more implementations as part of a shared action-reflection process.

We drew on notebook annotations and audio recordings to select quotations for the results and discussion. Quotes were selected strategically based on their capacity to illustrate the key insights and recurring patterns observed across settings, following an illustrative approach common to qualitative research (Glaser and Strauss 1967, p. xii; Breckenridge and Jones 2009, pp. 115–116). Although most quotations cited are from education settings, similar dynamics were observed in community and policy contexts. We selected quotations that most clearly illustrated broader findings. These serve as illustrative examples rather than exhaustive representations. To protect participant privacy, we provide minimal identifying details and instead emphasize the setting of each implementation.

## Reflections on implementing the council of care

### Transdisciplinarity insights

Transdisciplinary projects involve a range of actors, including both academic and nonacademic groups. The development and application of the Council of Care enabled us to navigate key potentials and challenges within such collaborations. Here, we discuss two main insights from our implementations: building trust and navigating power dynamics.

#### Facilitating the development of trust relationships

Throughout the Council’s sessions, we observed that some participants—particularly those who were typically shy or introverted—gradually became more open and vocal. This shift was evident across different settings, especially in the Local Community Setting. For example, in one session, a fisherman mimicked a hawk during the embodiment phase, and later shared how meaningful the experience had been for him. He expressed that he not only learned something but also felt that he could teach us, while having fun (Implementation 6, Local Community Setting). These moments highlight the role of trust in fostering participation, creativity, and emotional expression. They also taught about how long-term collaboration can create conditions for deeper engagement and taking risks/trying new approaches.

As discussed earlier, transdisciplinarity aims to foster dialogue across diverse sociocultural knowledge systems (Scholz and Steiner 2015, p. 522; Popa et al. 2015, p. 46; Bammer et al. 2020, p. 6; De La Rosa 2024, p. 698). Trust is widely recognized as a fundamental condition for these dialogues to occur meaningfully, allowing participants to express their experiences, needs, and fears (De La Rosa et al. 2024, p. 698; Staffa et al. 2022, p. 58). In our work, the Council of Care provided an environment where trust could be cultivated, enabling not only expression but also the collective discussion of concrete issues. This environment of openness, enjoyment, and reflection helps nurture relationships within the community and between participants and mediators. However, learning together also brings to the surface the matters of power.

#### The role of power

While facilitating the Council of Care, we noticed that the dynamics between participants were strongly influenced by the setting and pre-existing relationships. These relationships are permeated by power, which also plays a significant role in transdisciplinary collaboration (Turnhout et al. 2020, pp. 15–16; Hildyard et al. 2001, pp. 68–71), making it essential to understand how authority, representation, and voices manifest during collective processes.

During our implementations, power became visible in at least three ways: (a) participants’ interpretations of different roles or beings; (b) the perceived authority of the mediators; and (c) the influence of pre-existing social hierarchies between participants.

In the Education Settings, both the research team and other academics were often attentive to relational dynamics. This helped us decide who should be involved in the sessions and how to frame the engagements. We observed that the way participants interpreted certain beings—such as crabs, politicians, mangrove trees, or researchers—was shaped by how they perceived power. For instance, participants often described feeling powerless when embodying a crab or a mangrove, whereas roles such as policy-maker or researcher were sometimes experienced as carrying overwhelming responsibility. These dynamics were not just about the imagined beings—they also reflected participants’ lived histories and relationships, both inside and outside the Council.

Being able to read and respond to these power dynamics was only possible because, in some settings, such as Siribinha, we had already developed long-term trust relationships with participants. We emphasize the importance of integrating multiple methods to understand context beforehand. In one implementation, a participant offered this feedback: “Mediators should be careful in implementing this method in conflictive contexts; I worry that it can be dangerous in the sense of instigating more conflict” (Implementation 4, Education Setting). This feedback reinforced the importance of preparing and knowing the context and the people involved before facilitating potentially sensitive role-play and decision-making.

We also learned that facilitators must remain attentive to their own position and authority. A "know-it-all" stance, or inadvertently placing participants in the spotlight, can stifle openness. Power is not just a background issue; it shapes participation at every stage. In Siribinha, the Council of Care allowed us to observe how participants embodied other beings, interpreted each other’s roles, and negotiated collective responses to shared issues.

Another important insight was how the presence of specific individuals influenced the behavior of others or the group. Community roles and reputations influenced how people spoke, listened, and made decisions. These human-to-human dynamics were further complicated by the additional complexities of speaking as or for nonhuman beings—raising questions about representation and the limits of voice—which we further discuss in the following section. Going beyond the human–human dimension, we must also reflect on the more-than-human care relations that were evident during the Council of Care.

### Care insights

A transdisciplinary, participatory, and arts-based method that aims to foster transformation must integrate principles and practices of care (Sellberg et al. 2021, p. 301). In our experience implementing the Council of Care, two key dimensions of care emerged as central: empathy and paying attention.

#### Learning and practicing empathy

After choosing a being, participants are invited to engage in the Council through empathy, meaning putting themselves in the shoes of others. In this process, participants are encouraged to try to see with their own eyes from a position that is not their own (Biesta 2016, p. 187; Arendt 2003; Ormond and Vietti 2021, p. 536; Ressiore 2022), which can invite them to see a plurality of ways of being and caring in the world (Ressiore et al. 2024b, p. 18).

To support this process, mediators guide participants through the embodiment step, using questions such as the following: What kind of care do you require now? What kind of care do you give? Are you able to care for others? How do you care for yourself in this situation? (Annex 3). These prompts aim to foster both caring for and caring about, as they encourage reflexivity, needs, awareness, and relational responsibility (Tronto 2013, p. 22; Moriggi et al. 2020, pp. 4–7; Van de Pavert and Ressiore 2023, pp. 164–165).

However, empathy can be challenging—particularly when participants embody unfamiliar or nonhuman beings. Many expressed difficulty connecting with their chosen perspectives and were unsure how to act, speak, or feel. Comments like “I feel like I was not able to think like a mangrove” (Implementation 4, Education Setting) and “I didn't feel like I represented faithfully the crab” (Implementation 8, Education Setting) were common. One participant noted, “Being in the position of the nonhuman is very difficult. Taking the position of another agent is dangerous” (Implementation 4, Education Setting). Another reflected: “How to leave the human behind to embody a nonhuman?” (Implementation 8, Education Setting).

Despite this discomfort, many participants also noted a shift in perspective: “Although I believe it’s impossible to ‘become’ the other, it is possible to seek empathy, to try to understand the other and find something that connects us.” (Implementation 4, Education Setting). Other feedback emphasized how the method altered their view on care itself: “There is a strong care character to the tool. The method changed my view on care. Helped me see care from a different perspective.” (Implementation 5, Education Setting). Participants reflected not only on how they give and receive care in their daily lives, but also how care could be approached as collective, reciprocal, imaginative, and attentive practice in decision-making (Moriggi et al. 2020, pp. 4–7; Van de Pavert and Ressiore 2023, pp. 171–172).

#### Care as paying attention

Care emerged also as a practice of paying attention—to participants’ needs, to context, and to the tensions that arise during engagement. In feminist care theory, “care about” is often understood as giving attention to something or someone (Fisher and Tronto 1990, p. 35; Moriggi et al. 2020, p. 4). “Paying attention” can have an ethical connotation that prompts responsibility, response, and gratitude (Kimmerer 2014, p. 20; Krzywoszynska 2019, p. 663).

This attentiveness was essential for adapting the method to diverse groups and settings, including noticing what participants needed, selecting relevant topics, incorporating feedback, and adjusting facilitation accordingly. It also allowed us to notice moments of productive discomfort. As Drotbohm (2022) explains, care produces a tension that demands negotiation: “care is an uncomfortable lens through which to seriously analyze the contingent nature of life's transformations and persistent instabilities”.

The participants frequently expressed discomfort around not knowing enough about the beings they embodied, which challenged their capabilities or inabilities to empathize. This discomfort sometimes triggered vulnerability or uncertainty. However, we came to understand this not as failure but as potential. Following Santos (2021), we began to see discomfort as the soundtrack of change—a sign that assumptions were being challenged and that new relational understandings might emerge. Echoing Haraway (2016, p. 1), the Council of Care invited participants to “stay with the trouble,” and from that, new seeds of possibility were planted.

The participant’s feedback confirmed that this discomfort could be both generative and transformative: “It was intriguing to think how it is to be a crab. I never thought about it before, but now I’m very curious about it” (Implementation 3, Local Community Setting). Alternatively, “This exercise is great for embodying another being. I truly felt like I was that being. I felt the emotions.” (Implementation 2, Education Setting). This required us to think carefully about what a respectful exchange means in each context. We learned to attune to group boundaries and emotional dynamics, aiming to create a safe and inclusive atmosphere. As one participant noted: “You (mediators) radiated peacefulness; you were not hurrying or seemed stressed” (Implementation 1, Education Setting).

**Fig. 5:**
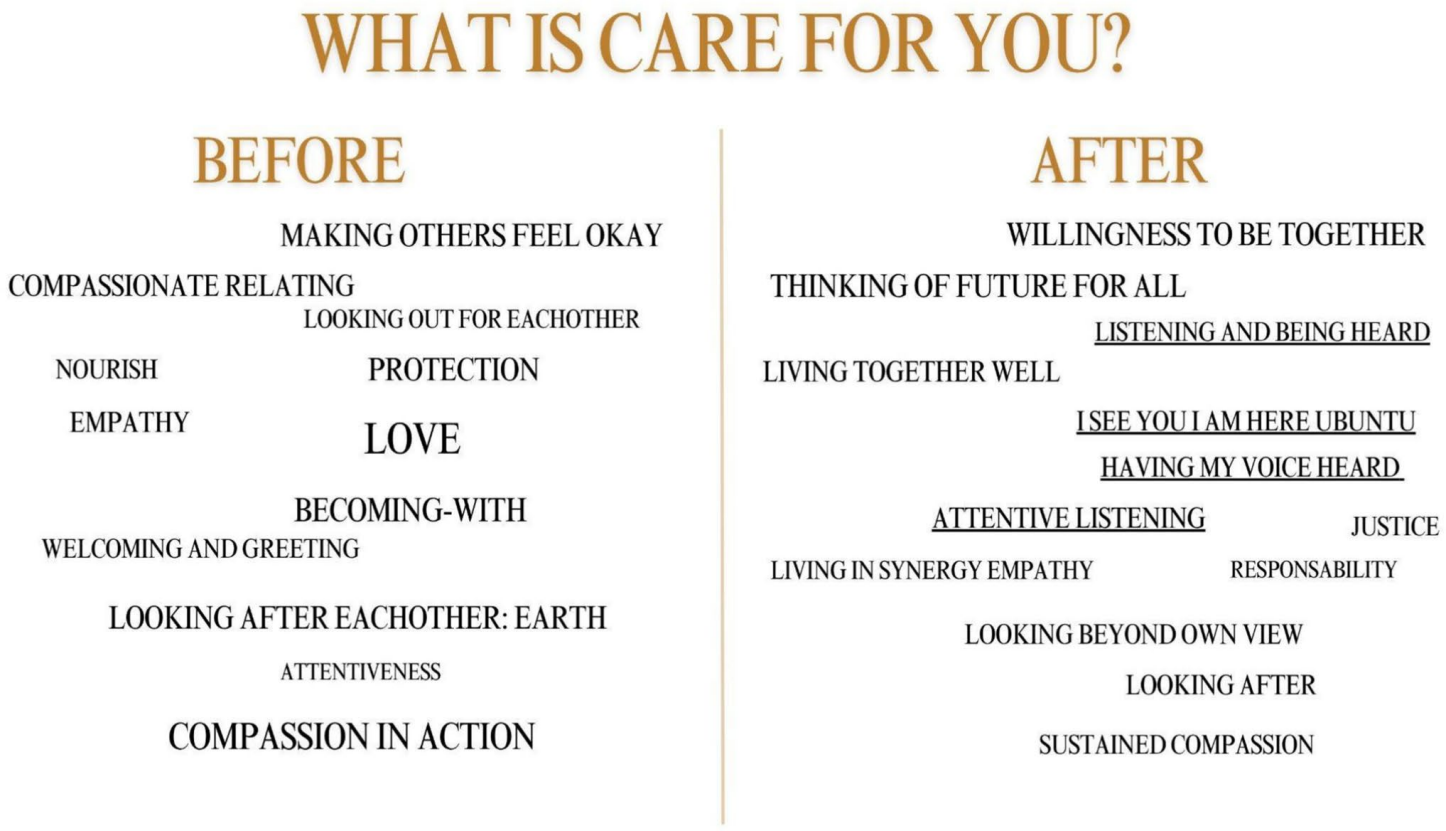
Word cloud, Implementation 5. We recreated the original word cloud to make it easier to read (Annex 6, Image 5). The underlined words highlight the aspects of listening in practicing collective care

In Implementation 5, the word clouds generated during the session revealed how participants associated care with listening and being attentive to others (Fig. 5). These became central themes, reinforcing the importance of being attentive to including marginalized perspectives and cultivating collective and caring decision-making.

### Transdisciplinary approach to collective care

Continuing from the previous discussion on care as paying attention, we reflect on how care, in the context of the Council of Care, extends beyond repairing a situation that is “wrong” or uncomfortable. It involves negotiating differences and adapting to relational complexities. Through implementation and based on feminist care theory, we were able to strengthen our view of more-than-human care as nonindividualistic. For example, implementations 3 and 6 in the Local Community Setting were not stand-alone workshops, thought and organized only by mediators, but rather a process involving several focus groups that encouraged participants to think and participate in choosing the issue to be discussed, as well as the concrete actions for and with the fishing village, among others.

While the method aims to foster collective processes by inviting “multiple intersecting identities into environmental decision-making” (Diver et al. 2024, p. 7), the participants also shared feelings about how challenging that can be when a large diversity of beings are being represented: “I learned that it was incredibly tempting to enter into a hopeless state in which nothing seemed possible when all human and nonhuman actors are present” (Implementation 2, Education Setting). This comment illustrates how participants articulated the difficulty of engaging with multiple intersecting perspectives during the Council, particularly when both human and nonhuman voices were involved.

From a transdisciplinary standpoint, implementing the Council of Care highlighted three key aspects that support collective care: interconnectedness, space, and the reflexive research process. The first aspect involves creating and encouraging communication in a safe environment. We guided participants to propose concrete actions to handle real-world problems. At this stage, some realized that these problems affect everyone differently. Understanding the interconnected relationships among participants became the first step toward developing policy recommendations that consider every being. This collective sense has the potential to foster an inclusive space where everyone is encouraged and supported to share their perspectives.

The second aspect pertains to the physical space where the implementation occurs. Feedback such as "The space in a plain white room with chairs, tables, and no possibility to move was not good" (Implementation 2, Education Setting) prompted us to rethink how space is utilized to create a welcoming atmosphere. Additionally, mediators work to maintain an open dialogue about the methodology, addressing the discomforts that arise and how to navigate them beyond the Council of Care. The environment can have a positive or negative impact on the sense of collectivity, as specific settings are more conducive to engagement, whereas others lead to disengagement (Pearson et al. 2018, p. 11).

The reflexive process was equally essential. Throughout the implementations, we engaged in cycles of reflection and adjustment, drawing on participant feedback and our own observations as mediators. These moments of self-assessment allowed us to remain attentive to shifting dynamics and supported the relational flexibility of the method.

Importantly, we recognize that care in transdisciplinary processes, when made collective, can risk erasing differences if not approached carefully. Following Giraud (2019, pp. 171–174), we consider that exclusion, refusal, and opposition can also play productive and creative roles. These forms of exclusion need to be made visible to foster a sense of responsibility and obligation toward them. For example, when policy-makers were represented in the Council, the responsibility and requests to act would often be strongly directed to them. At the same time, when they were not present, participants frequently felt that they were missing. In this sense, the Council also considers the tensions with ideals of universal inclusion, encouraging a careful and reflective engagement with boundaries and differences in each implementation, therefore aligning with the objectives of implementing the Council with a specific group.

However, even within these tensions, the Council revealed how collective orientations to care can still emerge—especially through structured moments of reflection. The collective aspect of care became particularly evident during the check-in and check-out exercises, when we asked participants to reflect on the following question: “What is care for you?” The answers tended to shift after the Council meeting took place. As shown in the word clouds (Annex 6), words from the check-in phase often reflected more individual or abstract notions of care. In contrast, in the check-out, terms such as “union”, “collective”, “integration”, “sharing”, “thinking about the other”, “together”, and “patience” appeared more frequently.

Centering on care, the mock decision-making process involves both human and nonhuman perspectives, inviting participants to move beyond “What do I need as an individual?” toward “What is possible to do for all (or as many as possible)?” This shift fosters collective thinking while revealing the challenges of collective action. Importantly, the Council of Care contributes to a transdisciplinary approach to collective care not by aiming for consensus, but by opening space for meaningful engagement across differences.

## Potentials and challenges for the decision-making process

Based on the theoretical and empirical analysis discussed in the preceding sections, Table 1 summarizes the key potentials and challenges of implementing the Council of Care in Local Community, Local Policy, and Education settings. It highlights setting-specific insights relevant to transdisciplinary and caring decision-making.

**Table 1:** Potential and challenges related to settings for the decision-making process

Setting	Potentials	Challenges
Local Community	1. Put oneself in the shoes of other community beings, possibly creating a sense of collective 2. Encourage communication and can organize decision-making 3. Helps to initiate a discussion about common goals 4. Contributes to identifying similar and diverging needs, desires, and fears 5. Include diverse methods and ways to exchange knowledge (using the body, arts, and expressions) productively; 6. Helps to understand the power dynamics between the participants 7. Facilitate the development of trusting relationships among the participants and between participants and mediators 8. Generate a fun and creative decision-making atmosphere	1. Time and resources needed to understand the local context thoroughly and be able to implement such a method 2. Not always easy for participants to feel comfortable sharing their knowledge and feelings 3. Often difficult (due to time and diverging viewpoints) to reach a consensus for a final policy brief 4. Need for follow-up meetings (time and resources) to achieve concrete actions 5. It can be a challenge or even undermine the researcher’s role when having to mediate the possible internal tensions and/or conflicts within the community (beyond the Council) 6. Not sufficient on its own to mediate or resolve deeper community conflicts; 7. We noticed sometimes a difficulty and discomfort in representing nonhumans
Local Policy	1. Put oneself in the place of other beings in the community could create a sense of collective needs and interests 2. The method can encourage communication 3. Helps to understand the power dynamics between the participants 4. Might encourage an inclusive and diverse decision-making process 5. Generate a fun and creative decision-making atmosphere	1. It can be challenging to schedule and find common availability 2. Not always easy for participants to feel comfortable sharing their knowledge and feelings 3. Develop collectively the necessity of marginalized human and nonhuman beings to be essential in the decision-making process
Education	1. Inspire other academics to work with arts-based methods, using multiple interdisciplinary methodologies, models, and tools 2. Inspire other academics to include nonhumans in their research 3. Put oneself in the place of other beings in communities that are facing environmental issues 4. Generate a fun and creative decision-making atmosphere	1. Challenge academics to rethink the agency of the nonhuman in research and decision-making 2. Make sure that the different activities are well integrated and coherent with the purpose 3. Create the right embodying atmosphere 4. Avoid essentializing being 5. We noticed sometimes a difficulty and discomfort of representing nonhumans;

While the Council of Care offers a valuable method for situated and imaginative engagement, it also reflects a deliberate focus on addressing real-world problems through simulated decision-making processes. The Council prioritizes affective engagement and relational learning. This focus makes it especially useful in contexts that value openness and co-creation, but also reveals limitations: the method may be less suited to institutional environments that demand quick or easily quantifiable results. Additionally, the need for skilled facilitation and the method’s arts-based and imaginative nature can challenge its integration in highly technical or hierarchical policy spaces. Importantly, the process does not usually conclude when the session ends—it requires ongoing engagement and time to build relationships, develop collective insights, and translate them into meaningful outcomes.

Our findings suggest that the difficulty participants experienced when attempting to speak for nonhuman beings reflects more than simply not knowing or the belief that it is not possible to represent them. It reveals deep structural asymmetries in power and knowledge production—one that continues to center humans as the primary agents of political participation (Meesters 2023, p. 18–19). It also reviews our often-inability to understand and voice the nonhuman subjectivities that are often deeply interlaced with our human ones (Bispo dos Santos 2023). Rather than overcoming this asymmetry, the Council of Care actually made it more visible, highlighting the profound challenges of genuinely including nonhuman perspectives in participatory processes. This resonates with insights from more-than-human participatory research (Bastian et al. 2017, pp. 6–8) and more-than-human care discussions, which emphasize that friction, difficulty, and uncertainty are not failures but rather essential features of transforming participatory methods to be more inclusive of nonhuman beings (Mol 2008, pp. 87–88; Puig de la Bellacasa, pp. 10 and 210; Ressiore et al. 2024b, p. 17; Ressiore et al 2024a, pp. 9–10). Addressing these asymmetries requires not only empathetic role-play but broader transformations in how agency, voice, and relational accountability are conceptualized and practiced in decision-making processes. We see this as a crucial direction for further research and methodological experimentation.

By further building upon the analysis presented in Table 1 and drawing on our insights into care and transdisciplinarity, we identified how the Council contributed to transdisciplinary and caring decision-making across various settings. An important nuance to consider in this discussion is how the various settings enabled us to achieve the results presented in multiple implementations. The Education Setting was extremely important in surfacing certain discussions that helped us improve the method, making us more prepared for fieldwork and implementation in the Local Community Setting. In turn, implementations in the Local Community and Local Policy Settings clarified the concrete potentials and limitations of the Council to engage with real-world problems and prompted context-sensitive adaptations.

**Fig. 6:**
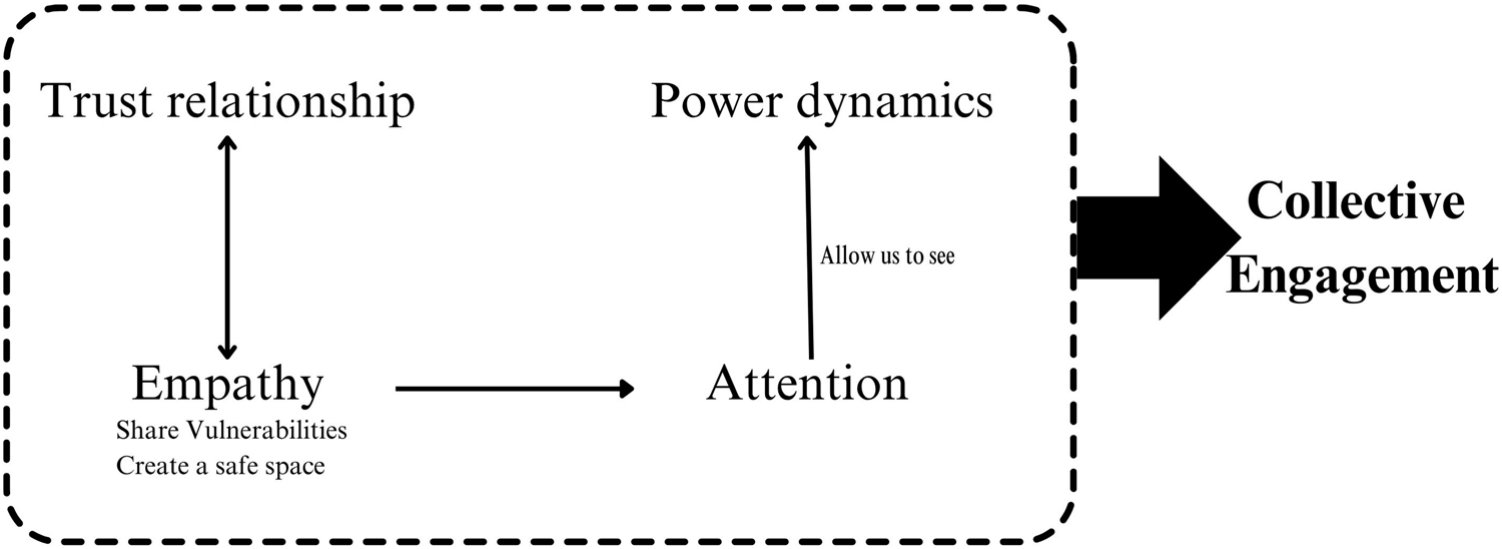
From Trust to Collective Engagement. Relation of key aspects of the Council of Care for Cultivating Collective Engagement in the Decision-Making Process

These distinct contributions allowed us to observe the importance of building trust relationships by creating a safe space to share our diverse vulnerabilities and foster a sense of empathy among participants. By practicing empathy with other beings, some participants and mediators were able to pay closer attention to nuances regarding needs, discomfort, and the implicit power dynamics that significantly influence processes and relationships beyond the Council of Care (Fig. 6).

In our perception, a combination of the factors mentioned above is related to cultivating a sense of collective, an essential element for the theory and practice of transdisciplinarity and care. As one participant noted in implementation 7, in the Local Policy Setting: “The methodology was beneficial to think from the perspective of other beings. It helped express connection and love for nature”. The Council of Care can foster collective awareness and actions—which, in the Siribinha context (De La Rosa et al. 2024, p. 701; Milberg Muñiz et al. 2025, p. 238) and other diverse contexts we have identified as essential steps for marginalized communities to advocate for their rights and aspirations among broader decision-making structures. This is especially important when the goal is to engage local communities in participatory processes that address complex socio-ecological challenges (Diver et al. 2024, p. 6).

## Toward a transdisciplinary and caring future

Through implementing and testing the method across various settings and issues—in both Dutch and Brazilian contexts—we gained valuable insights into transdisciplinary and caring methodological processes for engaging diverse actors in decision-making. This arts-based method is designed and adapted for Educational, Local Community, and Local Policy-Making settings. The method acknowledges that the problem affects everyone differently and that understanding the interconnected relationships between humans and nonhumans is crucial for creating policy recommendations that hear and engage with marginalized humans and represented nonhumans. While the method was developed in specific local settings, the challenges it aims to address—such as the marginalization of certain voices, the need for more participatory governance, and the limitations of dominant epistemologies—are widely shared across different regions and contexts. With the necessary integration of methods, the Council of Care offers a framework that can be adapted and applied in diverse socio-political and ecological settings and issues worldwide.

Our findings resonate with a growing international body of work that calls for transformative approaches to sustainability, participatory governance, and the inclusion of more-than-human perspectives in environmental and political processes (e.g., Contesse et al. 2021; West et al. 2024; Bastian et al. 2017; Meesters 2023; Rickard et al. 2024). Importantly, our approach contributes to ongoing global conversations about how to reimagine collective agency, relationality, and responsibility in response to complex planetary crises. As our implementations have shown, the Council of Care does not conclude at the end of the meeting; instead, it often leads to continuations or new beginnings.

Despite the discomfort and challenges that arise when building processes that engage with diverse and marginalized beings, we see these tensions as essential elements of an iterative cycle of learning, reflection, and transformation. In this sense, the Council of Care provides an adaptable and deeply relational approach that scholars and practitioners across regions might use to co-create more inclusive, caring, and responsive forms of decision-making. Inviting more researchers to engage and prototype with the potential of arts-based methods for decision-making processes can help to better understand, include, and transform the roles of marginalized human and nonhuman beings toward a more participatory and caring future.

## Supplementary Information

Below is the link to the electronic supplementary material.

Supplementary file1 (PDF 718 KB)

## Data Availability

Data are available on request due to privacy/ethical restrictions.

## Footnotes

1 While the authors, especially Adriana Ressiore have, extensively researched care and more-than-human care literature (Tronto 2013; Raghuram 2016; Barnes 2012; Bartos 2018; Cox 2010; Conradi 2015; Sellberg et al., 2021; Moriggi et al. 2020; Staffa et al. 2022; Mol and Hardon 2021; Care et al. 2021; Sandilands 1999), especially during the initial creation and imagination of the Council of Care, the work of Puig de la Bellacasa (2017) was the most influential literature on care we based ourselves. Her work was especially inspiring to think of care as something that was mutually happening between humans and nonhumans, but often also remaining invisible. Through the article it will be noticeable how other feminist care literature became also relevant for the discussion and conclusions.

2 Mourning involves participants expressing their grief and sorrow for the harm they have caused to the Earth (Macy and Brown 2014).

3 During the remembering part, participants are guided to reflect on their connections with the earth and other living beings. This aims at promoting belonging and unity with the natural world (Macy 2007).

4 Participants take on the roles of different nonhuman beings voicing and being heard, aiming to emphasize their interconnectedness of all life on Earth (Seed et al. 1988).

5 This stage is not part of the original book *Thinking Like a Mountain* but was later introduced to encourage participants to commit to real-life actions that help restore the Earth (Macy and Johnstone 2012). Some Council of All Beings practices do not always include this step. However, in the Council of Care, where we simulate a decision-making process, encouraging participants to consider actions that can be taken in the real world is a key aspect.

6 As it will become clear through the text: implementation refers to the actual application of the methodology. Settings refer to the groups of actors where the methodology has been implemented. Place refers to the place where the issue happened, for example, Siribinha. Issue: refer to the case presented to be discussed during the council’s discussion, for example, the oil spill.

7 Throughout the article, we occasionally refer to the Council of Care as “Council” for the sake of brevity and to avoid repetition.

8 The course is called Transformative and Participatory Qualitative Research Approaches and Methods (TPAR). It is held at Wageningen University in the Netherlands and was created and led by Meghann Ormond and Anke de Vrieze.

9 Model UN simulations engage students who are interested in learning more about the principles of the United Nations (UN) and how it functions (un.org, n.d.). The goal is to simulate real UN Council processes as realistically as possible, also serving to raise awareness about issues associated with International Relations (IR) and develop negotiation skills (Wesel 2000; Leib and Ruppel 2020).
