# Supplementary material for: Transdisciplinary care in practice: reflections from the Council of Care
Source: Socioecol Pract Res. 2025 Oct 27;7(4):499–517. doi: 10.1007/s42532-025-00229-w (PMC12727778; doi:10.1007/s42532-025-00229-w)
Supplement: Supplementary file 1 — Supplementary file1 (PDF 718 KB) [file 42532_2025_229_MOESM1_ESM.pdf]

The Council of Care for Transdisciplinary and Caring Decision-Making Processes

Annex 1: Contextualization for the Council of Care

(b) Contextualization

1. Place

The Council of Care implementations contextualize Siribinha as the location where the discussion takes place. To quickly introduce participants to a place rich in culture, biodiversity, and knowledge, mediators provide images showcasing the geographical location and biological aspects. Following this, the mediators introduce the issue that motivated the Council of Care's meeting.

2. Issue

The Council of Care implementations mentioned in section 3.2 address three main issues: the oil spill, the trash, and the fee (detailed below). The oil spill was the most frequently discussed issue across six implementations, and Table 2 illustrates how this information was presented to the participants. Media and academic articles have widely discussed the oil case, providing mediators with more materials to work with, including videos that help participants become acquainted with the place and issue. The trash and the fee were issues explicitly discussed in the local community setting because they were considered the most pressing issues at the time, as decided by the community. Although the oil spill significantly affected the community, during implementations 3 and 6, it was not considered an urgent concern.

The trash and fee issues required less contextualization, as these issues were implemented in the community itself. In this case, the participants were familiar with their context and the beings they represented. In these two cases, at least one member of each subgroup was found in Table 2.

2.1. Oil Spill

In the second semester of 2019, several fishing communities and coastal populations in Brazil faced what has become known as "the worst oil spill disaster not only in Brazilian history but also in any tropical coastal region worldwide" (Nasri Sissini et al. 2020 in Soares et al. 2022). The environmental tragedy affected eleven Brazilian states (Soares et al., 2022), spanning approximately 2,890 km of their coastline (Soares et al., 2022). The origin of this oil remains unclear and widely unknown (Soares et al., 2022). At the same time, media outlets have affirmed that the Brazilian Federal Police concluded their investigation and identified a Greek-flagged oil tanker as the source of the spill (G1 RN, 2021; Soares et al., 2022; Phys.org, 2021). The Greek-flagged oil tanker allegedly transported Venezuelan oil. However, it was not possible to verify this information, and researchers did not exclude other hypotheses (Zacharias et al., 2021a; Zacharias et al., 2021b; Soares et al., 2022).

The oil spill affected multiple living beings throughout Brazil. It affects several tropical ecosystems and their biodiversity, particularly estuarine water bodies, mangrove forests, seagrass meadows, beaches, tidal communities, sandstone reefs, and coral reefs (Magris & Giarrizzo, 2020; Soares et al., 2022). The oil reaches beaches, affecting tourism, turtles,

humpback whales, birds, and fishers (Uchôa, 2019). The oil suffocated the mangroves, killing the trees and the animals that inhabit and depend on them (Gragnani, 2019). In Siribinha, community members and researchers observed that the mangroves were severely affected, and tourism had been at a near-zero level for months. Community members highlighted that almost no emergency support was provided by local, state, or federal governments, and the village's inhabitants were cleaning the oil with their bare hands (Law, 2020; Milberg-Muñiz et al., 2024).

During the contextualization of the oil spill, the mediators presented two videos (Muniz, 2019a; Muniz, 2019b) for a crowdfunding campaign organized by members of our research team who work in Siribinha. These videos allowed participants to visualize the issue, see how it affects the local community, and understand how the researchers actively worked to help community members.

To simulate a democratic and representative political decision-making process, mediators divided beings (both human and nonhuman) into subgroups and contextualized each one. For example, beings such as crabs or mangrove trees were represented in the Council to advocate for the interests of their subgroup, the mangrove. In the case of Siribinha, there were five subgroups—see Table 2. Although the limitations of these roles and the nonhomogeneity within the groups were recognized, the aim was to include the broadest possible diversity of representatives.

Table 2: Contextualization of subgroups and their beings.

Subgroups	Beings	Contextualization provided	Oil impact
**Mangrove**	Mangrove tree;<br>Crabs;<br>Parakeet;<br>Hawk;<br>Itapicuru river;<br>River fish.	A place that includes a vast biodiversity and where primarily, but not exclusively, fisherwomen go to fish crabs.	Mangrove trees suffer from a lack of oxygen and can die. There are no more roots or seeds for animals to feed, and crabs are contaminated, if not dead.
**Beach**	Sea;<br>Turtle;<br>Sea fish;<br>Shark;<br>Humpback whale.	The beach attracts tourists.<br>It is also where many turtles come to lay their eggs every year; during summer, (mostly) fishermen fish on the sea; Humpback Whales reproduce on the coast (from August to November);	The oil left a toxic trail for thousands of kilometers along the beaches, degrading corals. This entails decades of contamination of the sea environment and people.
**Tourism/ Commerce**	Boat tour guide;<br>Little hotel owner;	Tourism is an essential source of income; it brings resources to hotel owners, boat tourist guides, and bar and restaurant owners.	The oil spill heavily impacted this sector. Reservations were cancelled at all the little hotels, many

	Little shop owner;<br><br>Bar owner;<br><br>Tourist.	Tourists who come to Siribinha are usually from lower or middle-income (no luxurious resorts or hotels).<br>There are no paved streets in the village, nor paved roads to get to the village.<br>Some members of this subgroup are interested in developing Siribinha, which would increase the number of tourists.<br>Like other nearby beaches, a gentrification process is likely to occur and is already underway at Siribinha.	owned by community members. Boat tour income was no longer generated; there were months with almost no tourists.<br>Emergency resources were provided only after a while and were not specific to this sector, as they were for the village's inhabitants.
**Fishers**	Fishermen;<br><br>Fisherwomen/shellfish gatherers;<br><br>Local school teacher;<br><br>Child/Teenager.	Fishing is Siribinha's primary source of income; both fishermen and fisherwomen possess artisanal knowledge and practices.<br>Some fishing activities are gendered, although this is not a universal rule; we have observed men fishing in the mangrove as well as women fishing from boats in the river and the sea.<br>Most women fish in the mangrove or with their husbands or family members in the river, while fishermen fish mostly in the river and the sea.	They could not sell their fish and shellfish and were afraid of eating what they had caught, but they did not have many other options. For a while, they had no emergency resource support from the local, state, or federal government. Many were cleaning the oil from mangroves and beaches with their bare hands.
**Researchers**	Brazilian man and experienced researcher.<br><br>Brazilian woman and junior researcher.<br><br>A foreign and junior woman researcher.	Research projects initiated in 2016 comprise a multidisciplinary group of researchers. Many Siribinha inhabitants are reluctant to work with researchers (feeling abandoned).<br>Other inhabitants have worked with the researchers from the start and value the research projects.<br>There seems to be no clear community leadership and many internal conflicts within Siribinha.<br>Researchers, based on participatory workshops, have identified the lack of unity as one of the main complaints of community members.<br>The researchers have organized clean-up days in partnership with community members.	During the oil spill, researchers organized an international crowdfunding campaign to gather emergency resources for the community. The cash was converted into food baskets for every family.
**Policy-maker**	The head of the Conde Municipal	The head of the Secretariat is not from Siribinha village but from	During the oil spill, they sent bags and

	Secretariat of Environment and Economic Development;<br><br>An employee of Conde’s Municipal Secretariat of Environment and Economic Development;	Conde, the central city in the municipality.<br>The character is an experienced man who has completed a graduate study. He was one of the responsible people for creating a Conservation Area in Siribinha, which generated several conflicts and possible benefits.<br>There is a lack of trust from Siribinha inhabitants regarding almost all policy-makers from Conde.	gloves for the inhabitants to use while cleaning the oil. This was done only at the request of Siribinha community members.

Description: The names are listed in column 2. In smaller group implementations, we typically use only the first or second beings on the list. When there were more participants, we included additional beings listed. In general, we aim to have an equal or greater number of nonhuman beings than humans do. The level of contextualization, as well as the explanation of the oil's impact, depends on the time available for implementing the Council. If time is limited, we may send information to participants in advance or provide printed briefing cards that allow them to quickly understand and embody their character. With more time, we present each subgroup in detail, including their context, their beings, and how they were affected by the oil.

2.2. Trash

During Gabriela’s master's research and fieldwork (2018–2020), which focused on creating a bottom-up conservation unit in the community, key participatory findings emerged (Anonymised-for-review, 2020). These included a perceived lack of unity among community members and the issue of solid waste accumulating on beaches and in mangroves. Community members attending the workshops proposed and organized clean-up days to address both concerns. In Implementation 3, we held a workshop beforehand to identify issues the community wanted to address with us, and once again, trash was highlighted as a key concern. Since they chose the issue themselves, little contextualization was needed.

2.3. Fee

In 2021, a fee was introduced in Siribinha for access to *Boca da Barra*—the beach where the river meets the sea—the village's most popular tourist spot. The area was designated a natural monument (a specific type of Brazilian protected area). This decision surprised community members, as they were not consulted and felt that the process was top-down. They were unclear about the fee's purpose and where the funds would go, and they expressed concerns about its negative impact on tourism. Given the significance of this issue and its frequent mention, together with the community, we decided to discuss it during Implementation 6. Our main goal was to reflect on how the community might have engaged differently if the process had been participatory.

References

De La Rosa, G. (2020) Planejamento Estratégico e Conservação com base em participação comunitária no Estuário do Itapicuru, Conde-BA. Master thesis, Universidade Federal da Bahia.

G1 RN (2021) PF conclui investigação e diz que navio grego foi responsável por derramamento de óleo que atingiu litoral brasileiro. Portal G1. https://g1.globo.com/rn/rio-grande-do-norte/noticia/2021/12/02/pf-conclui-investigacao-e-diz-que-navio-grego-foi-responsavel-por-derramamento-de-oleo-que-atingiu-litoral-brasileiro.ghtml

Gragnani, J., 2019. Essenciais para o planeta, manguezais no Nordeste são 'sufocados' por petróleo - BBC News Brasil. [online] BBC News Brasil. https://www.bbc.com/portuguese/brasil-50132770.

Law, J., 2020. Full impact of mysterious Brazil oil spill remains unknown. [online] BirdLife. https://www.birdlife.org/worldwide/news/full-impact-mysterious-brazil-oil-spill-remains-unknown.

Magris, R. A., & Giarrizzo, T. (2020). Mysterious oil spill in the Atlantic Ocean threatens marine biodiversity and local people in Brazil. Marine pollution bulletin, 153, 110961.

Milberg-Muñiz, E., Ludwig, D., & El-Hani, C. N. (2024). Research as a Mangrove: Emancipatory Science and the Messy Reality of Transdisciplinarity. International Review of Qualitative Research, 0(0). https://doi.org/10.1177/19408447241260446

Muniz, E. M. (2019a) The communities of Itapicuru River estuary are asking for your help! [Video]. YouTube. https://www.youtube.com/watch?v=gfSR8jzCT88&ab_channel=EM.Muniz

Muniz, E. M. (2019b) Campanha de arrecadação. [Video]. YouTube. https://www.youtube.com/watch?v=laqbRRuNZCQ&t=6s&ab_channel=EM.Muniz

Nasri Sissini, M., Berchez, F., Hall-Spencer, J., Ghilardi-Lopes, N., Carvalho, V. F., Schubert, N., ... & Horta, P. A. (2020). Brazil oil spill response: Protect rhodolith beds. *Science*, 367(6474), 156-156.

Phys.org (2021) Greek-flagged ship caused Brazil mystery 2019 oil slick: police. Earth/Environment. DECEMBER 3, 2021. https://phys.org/news/2021-12-greek-flagged-ship-brazil-mystery-oil.html [accessed 13/11/2023]

Soares, M. O., Teixeira, C. E. P., Bezerra, L. E. A., Rabelo, E. F., Castro, I. B., & Cavalcante, R. M. (2022). The most extensive oil spill registered in tropical oceans (Brazil): the balance sheet of a disaster. Environmental Science and Pollution Research, 29(13), 19869-19877.

Soares, M. O., Teixeira, C. E. P., Bezerra, L. E. A., Rabelo, E. F., Castro, I. B., & Cavalcante, R. M. (2022). The most extensive oil spill registered in tropical oceans (Brazil): the balance sheet of a disaster. Environmental Science and Pollution Research, 29(13), 19869-19877.

Uchôa, V. (2019). Danos do óleo no litoral do Nordeste vão durar décadas, dizem oceanógrafos - BBC News Brasil. [online] BBC News Brasil. Available at: <https://www.bbc.com/portuguese/brasil-50131560> [Accessed 2 March 2021].

Zacharias DC, Gama CM, Fornaro A (2021a) Mysterious oil spill on Brazilian coast: analysis and estimates. Mar Pollut Bull 165:112125. https://doi.org/10.1016/j.marpolbul.2021.112125

Zacharias DC, Gama CM, Harari J, Rocha RP, Fornaro A (2021b) Mysterious oil spill on the Brazilian coast – part 2: a probabilistic approach to fill gaps of uncertainties. Mar Pollut Bull 173:113085. https://doi.org/10.1016/j.marpolbul.2021.113085

Annex 2: Council of Care Structure

In Table 3 below, we provide a description, suggested adaptations, recommended duration, and the relevance of each step in the Council of Care structure as we have developed it.

Table 3: Council of Care Structure: Description, Adaptations, and Relevance.

ID	Suggested Time	Steps	Description	Adaptations	Relevance
(a)	5 min	What is care for you?	The check-in exercise uses the prompt question: What is care for you? While participants say words that come to mind, mediators write them down on a board.	Online tools can also be used. In this case, participants insert the word that comes to mind on their smartphones, creating a word cloud.<br><br>An object brought from home that represents care for each participant can also be used.<br><br>Mediators can use a board or paper to write down words.<br><br>See examples in Annex 5	Serves to warm up, connect, and convene (Pearson et al., 2018)<br>Prompt participants to start the session by considering the many meanings of care.
(b)	7 min	Contextualization	This is a crucial moment when participants can slowly immerse themselves in a specific place and issue.<br>Provide as much information as necessary for a rich Council discussion. Examples used in our implementations are in Annex 1.	Using a PowerPoint presentation - it can contain images, videos, data, etc.<br><br>Or introduce the setting and the issue orally.	Allows participants to align their narratives and embodiment with the place and issue.
(c)	Embodiment				
(c.1)	3 min	Picking the being	Mediators bring badges with pictures and names of the beings.<br>Participants get up, choose their badge,	When conducted online, the being's image can be used as the background in the video call. Participants should also change their	Allows people to choose beings they might identify with or are curious about embodying.<br>Mediators stimulate a democratic process

			and select who they will be from a table. Refer to Annex 1, Table 2, for beings.	names to match the being's name.	for choosing the being.
(c.2)	5 min	Breathing exercise & meditation	Short breathing exercises with eyes closed. Mediators guide participants in the process of embodying another being.<br>For the text used in the embodiment, refer to Annex 3.	Within the Local Community Setting, mediators learned that meditation and breathing do not work as well as more interactive practices. Refer to c.4.<br><br>The breathing/meditation and embodiment text (Annex 3, Box 1) can be rearranged/rewritten according to the mediator's preference and participants' needs.	Create the space and the mindset to put yourself in someone else's shoes.
(c.3)	5 min	Evoke the senses	Mediators invite participants to write down their feelings, thoughts, and concerns. They are also free to draw and prepare their opening speeches.	Depending on the setting, including background sounds such as those of a forest and birds, can aid in the embodiment process. In our case, mediators like to use ocean background noise.<br><br>Council could be done without this step, and only c.4.	Introspective time to embody someone else and reflect on their being's needs and positionality.
(c.4)	7 min	Introduce yourself	Mediators invite beings to stand up and start moving as their authentic selves; when mediators clap, they must introduce themselves (as that being) to someone next to them. A few rounds of one-on-one introductions take place.	The council could be conducted without this step, and only sections c.1, c.2, and c.3.	Feeling, speaking, and moving as a being makes the embodiment process more interactive and dynamic. It can also be an opportunity to meet and make allies.

(d)	Council Discussion				
(d.1)	13 min	Opening of the Council Meeting	Participants are invited to sit in a circle (see Illustration 1).<br>Mediators declare the Council of Care meeting officially open.<br>A round of opening speeches takes place.	Can be done with tables & chairs or only with chairs.	Participants will set the tone of the discussion with their opening speeches. They can propose discussion topics, accuse other council members, ask for help, say what kind of care they require, etc.
(d.2)	15 min	Council's Meeting	Participants are stimulated to continue discussing.<br><br>If needed, mediators provide prompt questions (refer to Annex 4, Box 2)<br><br>Mediators should keep a pen and paper to annotate the order of the speakers' list.<br><br>Refer to the being's badge name when calling them to speak.	Hand gestures could be used to mediate and make the council's discussion more vivid; see image 2, Annex 4.	Simulating a formal council meeting where different representatives are invited to represent the interests of their subgroups, participants can slowly assess the complexity of the collective and caring decision-making process.
(d.3)	14 min	Receive economic resources to solve the issue	Mediators announce that resources have arrived and can be used to solve the issue being discussed.	Council can be done without this step, resulting in more time for d.2	Serves to question whether or not economic resources can help them to find common solutions.
(d.4)	10 min	Policy Brief	Mediators stimulate participants to develop policy recommendations or a common document that can be used to solve the issue at hand.	Mediators can write down proposals.<br><br>Participants may take the lead in preparing a document.	Provide a concrete follow-up resulting from the discussion.
(d.5)	2 min	Close the session and disembodiment	This is when the mediators declare the session closed. After a brief breathing exercise, they invite participants to remove their badges and leave their characters behind.	If time allows, mediators may take more time to engage in interactive exercises, drawing or writing, or longer meditation.	Provides a moment for disembodiment, which is essential for leaving the chosen being behind and feeling like oneself again.

(e)	10 min	Check-out: What is care for you? Did something change?	After a long discussion and embodying another being, mediators ask participants again: What is care for you? Did something change?	Repeat the same method as the chosen option from (a) Check-in.	This is relevant for closing with reflexivity. By placing the word cloud from the check-in (a) in contrast with the words emerging in this session, mediators and participants can identify shifts in mentality. This applies to both group and individual perspectives on care as a practice and as a concept.
(f)	18 min	Feedback and harvest	Open dialogue between participants and mediators.<br><br>See Box 3, Annex 5, for prompt questions related to feedback and harvest.	It can be done in a round of impressions where all participants have a moment to share.<br><br>Or mediators can spontaneously open up for participants to speak up.	A moment for mediators to collect feedback on the experience and the methods. This serves as inspiration for future adaptations.<br>It also consolidates and summarizes lessons learned, reflections, and insights (Pearson et al., 2018).
Total	120 min				

Annex 3: Embodiment

After introducing the place and the issue, mediators start the embodiment process. In the Council of Care, representatives from all subgroups are present to advocate for their interests and needs, and to speak on behalf of those who are not present.

(c.2) Breathing exercise & meditation

This is a moment to create the space and mindset to embody another being. The mediators refer to the text in Box 1. Small adaptations and translations were made to the main text, depending on the location and issue being discussed.

Now, we will complete our journey to arrive at (insert chosen place). I will ask you to try to leave your current self behind and start to imagine that you are the being you just chose.

Breathing Meditation

Sit comfortably. If possible, align your spine and then close your eyes. Your arms should be relaxed along your body, and your hands should be placed on your legs with the palms facing up.

Breathe naturally through your nose. Slowly imagine that your torso is like a cup. When you inhale, bring the air to your belly, then to your ribs, and let the air fill your chest. When you exhale, do the opposite. Therefore, empty first your chest, second your ribs, and then your belly. Try to bring your belly button as close to your back as possible. When you inhale, you fill the cup (belly, ribs, and chest), and when you exhale, you are emptying the same cup (chest, ribs, and belly). Keep going, own your own time.
It is normal that during your breath, many thoughts come to your mind; do not worry about it. Just let them come and go. Keep your attention on your breath now.

Slowly start to feel your heart. Pay attention to your heartbeats. Realize how this organ is powerful and fragile at the same time, and notice all the life that comes from each beat. Now, let your breath flow naturally. No need to control it. Slowly notice that within your heartbeats, there is a light that starts to shine; it becomes stronger with each beat. This light is now crossing the borders of your heart, occupying the entire space of your body, crossing the borders of your physical body, and letting your energy get bigger and bigger.

Embodiment Text

With your eyes closed, start to imagine and hear the ocean waves. You hear some birds far away. The wind is blowing into the coconut trees. You smell the sea, and you feel sand/earth under you. You hear some people chatting nearby. You feel the warmth, approximately 30 degrees, and the breeze touches you. The sun shines, and the sky is blue with almost no clouds. You are gathered under a shade.

(WAIT)

Now imagine how living in this being body feels: what is your shape? What does your skin feel like? Do you have any skin? What kind of voice or noise do you make? How much

space do you take up? (WAIT) How do you take notice of what is around you? How do you move, or how are you moved by other forces? (WAIT)

How do you feel at this moment? Are you afraid of some of the beings next to you on the Council? Do you already have some connections, friendships, and partnerships with some of them? (WAIT) Are you worried? Do you have oil on you? How is the oil spill affecting you?

(WAIT)

What kind of care do you require now? What kind of care do you give? Are you able to care for or give care to others? (WAIT) How do you care for yourself in this situation? What do you care about at this moment?

Please keep your eyes closed.

Slowly rub one hand against the other until you have some warmth within them. When you feel a comfortable temperature, make two shells with your hands and put them in front of your eyes. Slowly start to blink your eyes and look at your palms until your eyes are completely open. Then, take off your hands and look at the space and the beings around you.

Welcome!

Box 1: Council of Care Embodiment and Meditation Text

Annex 4: Council Discussion

(d.2) Council's Meeting

The mediators welcome beings and declare the Council of Care meeting officially open. Mediators may introduce hand gestures (Image 1) to mediate the discussion.

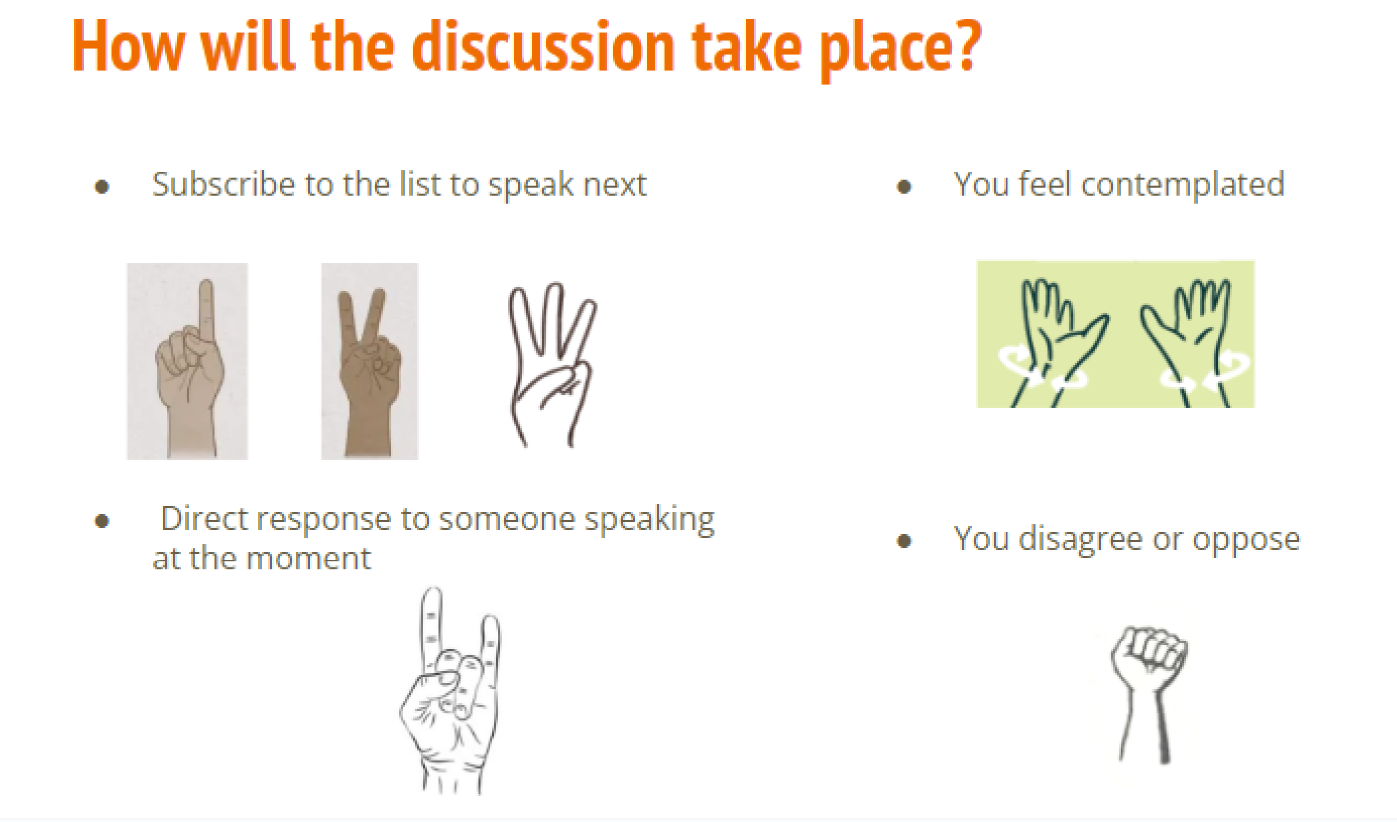

Image 1: Hand gestures. These can be options to mediate the discussion. Mediators can combine gestures with a list of subscriptions. The images were taken from Wall-Skills, Seed of Change organization and free picture websites.

After opening, the mediators give every participant the floor an opening speech.

If participants have difficulty engaging in the discussion, the mediators can bring prompt questions to stimulate the debate. This can be done with PowerPoint or orally. See box 2 for examples.

- How is the oil situation affecting you?
- How is it affecting your community?
- Can you find things/interests you all have in common? What don't you have in common?
- What are the next steps, in your opinion?
- How could a balance be restored? Were we actually in balance before?
- Do you feel like your needs are being heard?
- Do you feel like the policymaker cares for/with you? How do you think they must care about you?

- Could you collectively develop a policy brief?

Box 2: Council's Discussion Prompt Questions. The questions may stay on the screen to engage participants.

(d.3) Receive economic resources to solve this issue

The first two implementations did not contain this part of the method. The mediators decided to bring resources to test whether they would fuel or motivate the conversation. This aims to determine whether resources make the discussion more solution-oriented. The mediators bring the news after approximately 15 minutes of the Council's meeting. The amount of resources made available is typically R$100,000.00 (BRL) or € 100,000.00.

(d.4) Policy Brief

Usually, 10 minutes before mediators close the Council, if it is not already happening organically, it is important to stimulate participants to come up with proposals, suggestions, or a policy brief that could lead to concrete actions.

Annex 5: Feedback and Harvest

(f) Feedback and Harvest

This is a moment to reflect on what was experienced. It is also a moment when mediators invite feedback on the mediation, including what could have been done differently, potential adaptations, and so on. See Box 3 for prompt questions related to feedback and harvesting. This is a crucial moment where participants share insights from their experiences; some identify with or disagree with one another. It has also been essential for mediators to reflect, improve, and modify the implementations.

- What methods worked well?
- Which moments did you have fun with and which ones not so much?
- How was the structure of the experience?
- What parts could be improved?
- How did we do as facilitators?

Box 3: Feedback and Harvest Prompt Questions

Annex 6: Word Clouds

See the photos and screenshots below, which show the word cloud responses from (a) check-in and (e) check-out, captured before and after the Council of Care session. We have highlighted words that reflect themes of union, collectivity, collaboration, patience, and related forms of shared care.

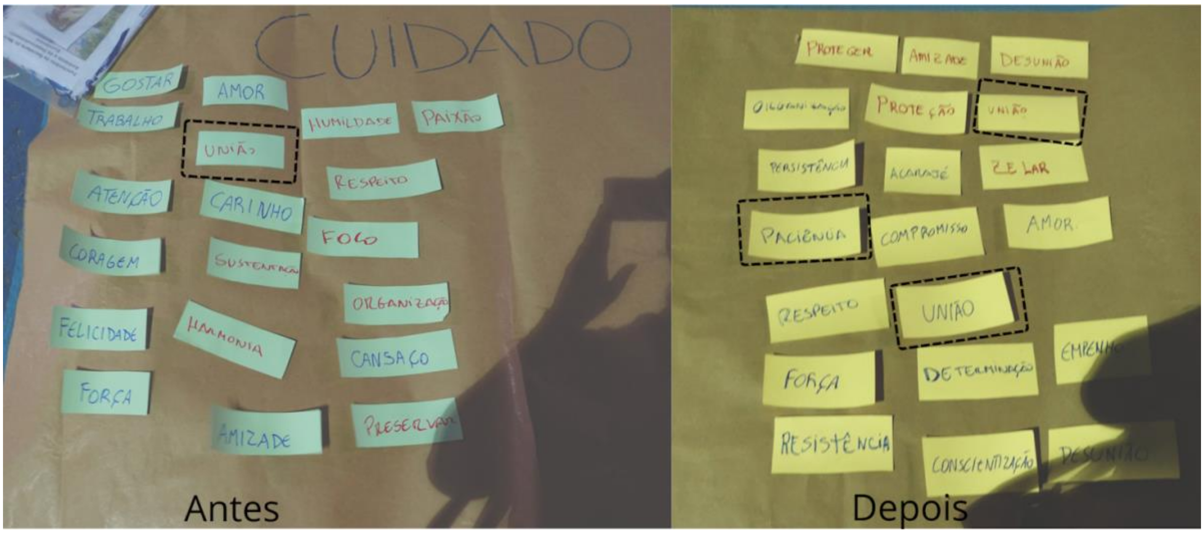

Image 2: Implementation number 3 - *Community Setting*, fieldwork in Siribinha 2022

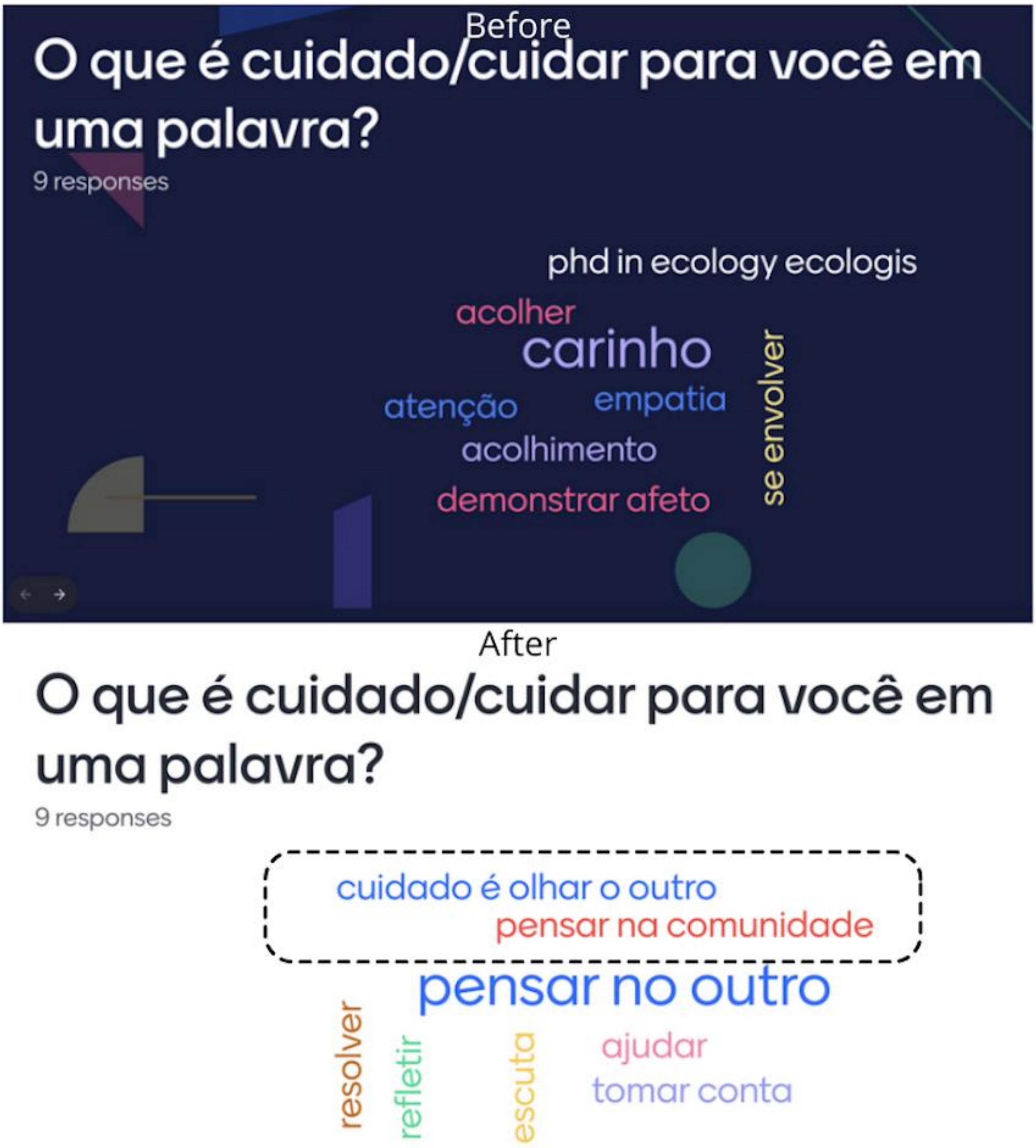

Image 3: Implementation number 4 - *Education Setting,* Laboratório de Ensino, Filosofia e História da Biologia (LEFHBio) - in English: Teaching, Philosophy and History of Biology Laboratory

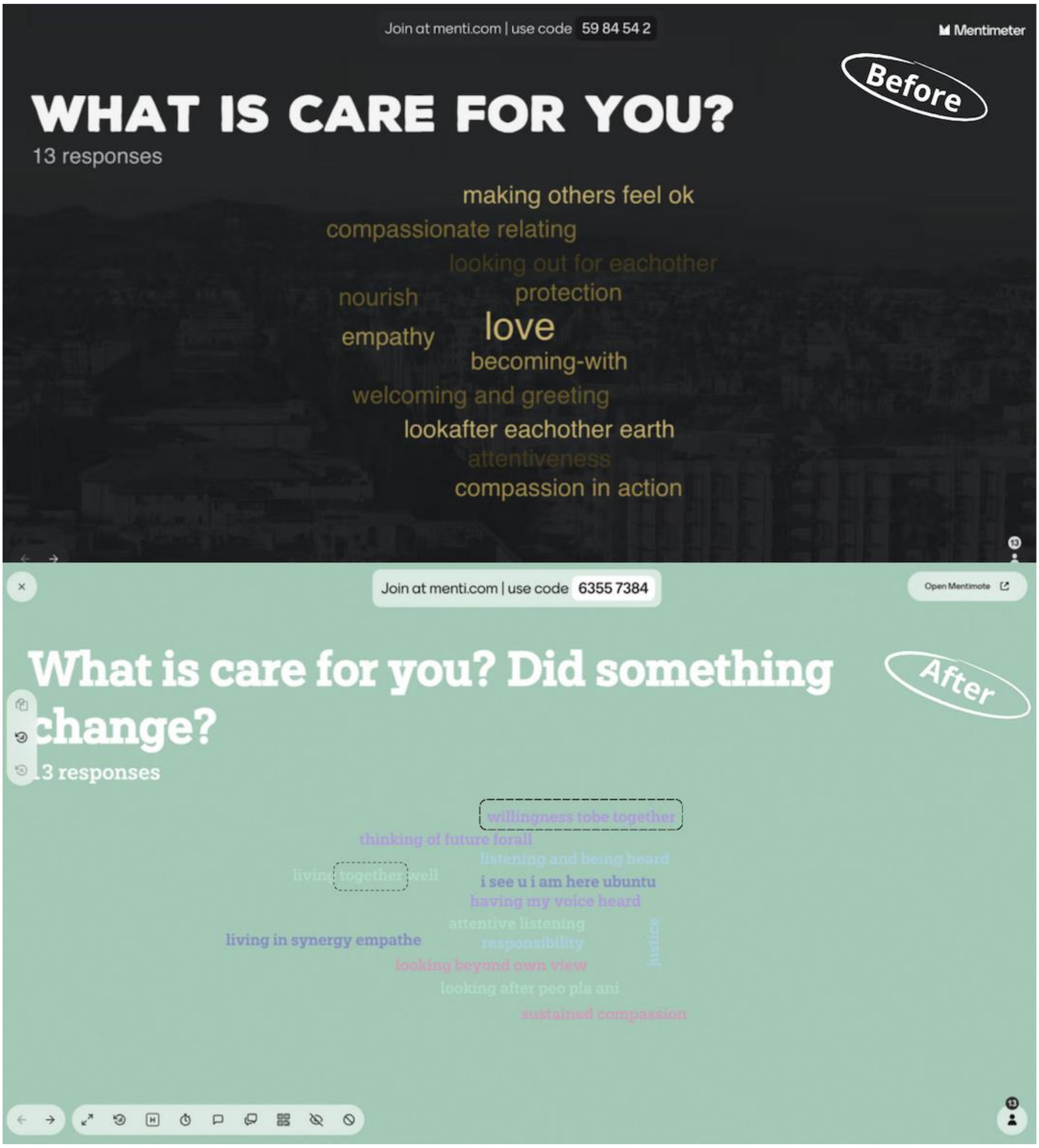

Image 4: Implementation number 5 - *Education Setting*, Transformative Learning Hub, Wageningen University

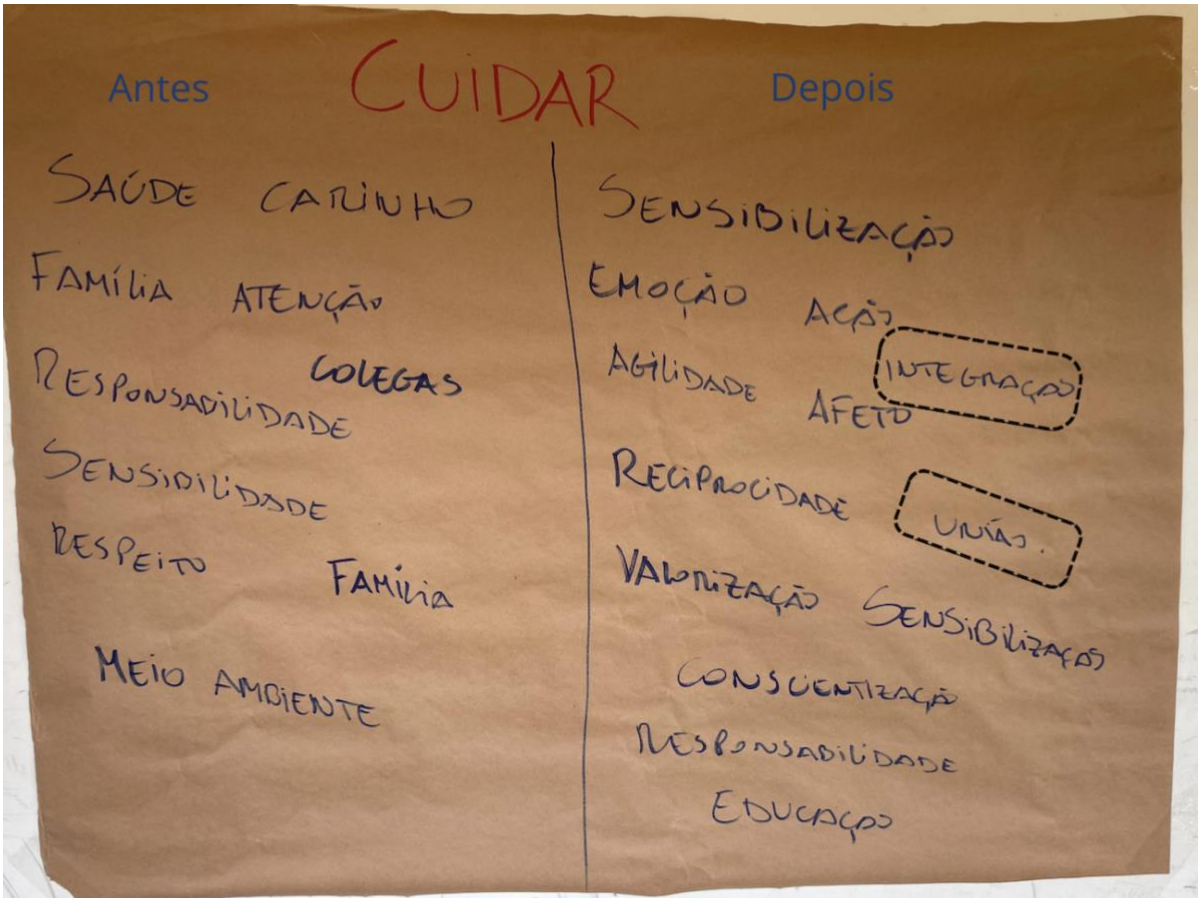

Image 5: Implementation number 7 - *Local Policy Setting*, Conde's Municipal Secretariat of the Environment and Economic Development

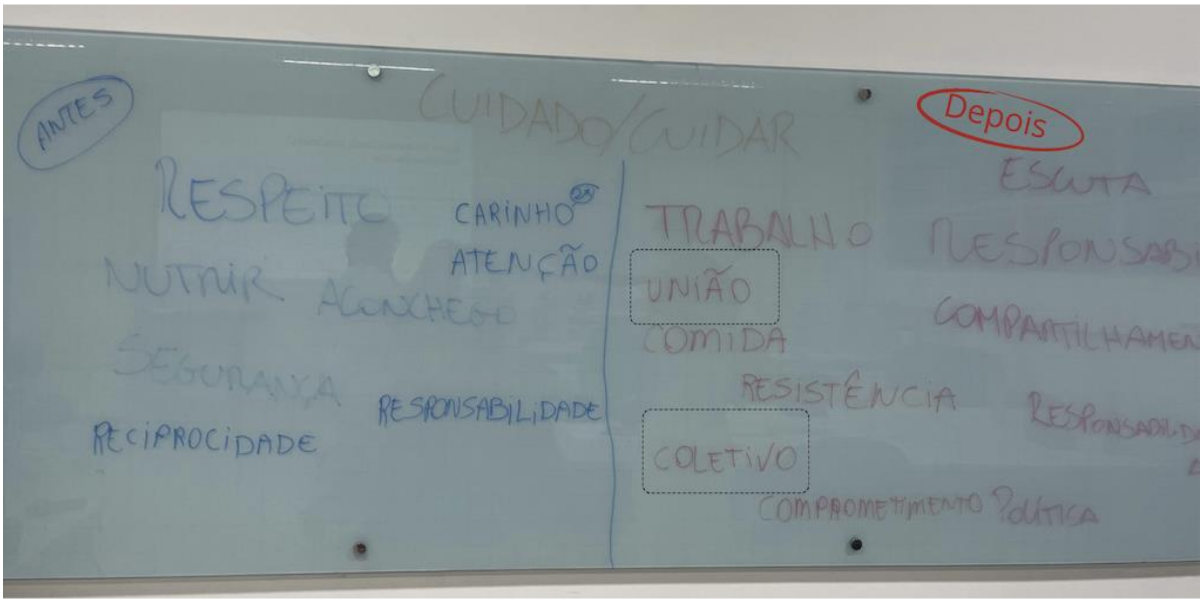

Image 6: Implementation number 8 - *Education setting,* Participatory methodologies: dialogue of knowledge and environmental conservation. Course created and taught by Adriana Ressiore, Gabriela De La Rosa & Juliana Fonseca at UFBA.
